# Insulin receptor substrate 2 (IRS2) confers resistance to PI3K pathway inhibition in *PIK3CA* mutant breast cancer

**DOI:** 10.1016/j.jbc.2026.113426

**Published:** 2026-08-10

**Authors:** Michael W. Lero, Jennifer S. Morgan, Michael-Anthony Card, Lihua Julie Zhu, Junhui Li, Rui Li, Quyen Thu Bui, Emma Mohlmann, Hira L. Goel, Leslie M. Shaw

**Affiliations:** Department of Molecular, Cell & Cancer Biology, University of Massachusetts Chan Medical School, Worcester, Massachusetts, USA

**Keywords:** AKT, breast cancer, cell death, cell proliferation, cell viability, insulin receptor substrate 1 (IRS1), IRS2, PI3K, *PIK3CA*

## Abstract

Activating mutations in phosphatidylinositol-3 kinase (PI3K) are one of the most frequent mutations in breast cancer and are associated with worse patient outcomes in many breast cancer subtypes. Despite intense interest, cancer treatments that target the PI3K pathway have been only modestly effective due to intrinsic and acquired resistance mechanisms which reactivate PI3K signaling. Here, we characterize a feedback mechanism by which PI3K pathway inhibitors increase insulin receptor substrate 2 (IRS2) abundance and demonstrate the role of IRS2 in promoting resistance to these drugs. In *PIK3CA* mutant breast tumors and cell lines, there is a significant reduction in IRS2 mRNA and protein abundance which is reversed by PI3K pathway inhibition and mediated by the transcription factors FOXO1 and FOXO3. *PIK3CA* mutations do not alter IRS1 expression. IRS2 confers resistance to PI3K pathway inhibition by sustaining PI3K signaling in *PIK3CA* mutant, but not WT breast cancer cells. Increased IRS2 abundance also correlates with PI3K pathway inhibitor resistance across *PIK3CA* mutant cancer cell lines from a variety of tissues. The clinical relevance of these findings is highlighted by the frequency of PI3K mutations in cancer and the identification of a new target to address the challenges associated with prior efforts to block the reactivation of PI3K signaling during PI3K inhibition.

There continues to be intense interest in understanding how phosphatidylinositol-3 kinase (PI3K) contributes to tumorigenesis and how it can be exploited therapeutically. *PIK3CA*, which encodes the p110α catalytic subunit of PI3K, is frequently mutated in human cancer, and is the second most commonly mutated gene in breast cancer (1). Three hotspot mutations, E545K, H1047R, and H1047L, account for 56% of all PI3K mutations in breast cancer (2). These activating mutations cause constitutive PI3K signaling, leading to growth-factor independent cell proliferation, increased cell survival and invasion, and generally worse clinical outcomes compared to patients with *PIK3CA* WT tumors (3, 4, 5, 6, 7, 8, 9, 10, 11, 12, 13). Two PI3K inhibitors have been shown to extend progression free survival in select *PIK3CA* mutant breast cancers and have received FDA approval (14, 15) (https://www.fda.gov/drugs/resources-information-approved-drugs/fda-approves-alpelisib-metastatic-breast-cancer, https://www.fda.gov/drugs/resources-information-approved-drugs/fda-approves-inavolisib-palbociclib-and-fulvestrant-endocrine-resistant-pik3ca-mutated-hr-positive). However, acquired and intrinsic resistance mechanisms significantly limit the clinical efficacy of these drugs.

Many clinically observed mechanisms of acquired resistance to PI3K inhibitors cause reactivation of PI3K signaling, either through secondary *PIK3CA* mutations, *AKT1* activating mutations, *PTEN* deletions, or *PTEN* loss of function mutations (16, 17, 18). Even in the absence of genomic alterations, PI3K pathway inhibitors elicit rapid intrinsic resistance mechanisms that reactivate PI3K signaling and limit drug efficacy. Because of the role of PI3K in glucose uptake and metabolism, PI3K inhibitors raise blood glucose levels, which leads to increased pancreatic insulin production and elevated blood insulin levels (19, 20). This excess blood insulin has been implicated in signaling through the insulin receptor to reactivate the PI3K pathway and promote tumor growth during PI3K inhibition in murine models of cancer (19, 21). PI3K pathway inhibitors also relieve negative feedback mechanisms that would otherwise limit PI3K signaling. This leads to many changes, including upregulation of the insulin-like growth factor 1 receptor (IGF1R), which reactivates the PI3K pathway. Interruption of this upstream IGF1R signaling has been shown to sensitize cancer to PI3K and AKT inhibition *in vivo* (22, 23, 24, 25, 26, 27, 28, 29, 30). Despite the fact that clinical PI3K-IGF1R treatment combinations have been discontinued because of concerns with side effects, these observations highlight the importance of both receptor-dependent and downstream reactivation of PI3K signaling in mediating resistance to PI3K inhibition (https://clinicaltrials.gov/study/NCT01708161).

This study was motivated by our interest in the potential contribution of the insulin receptor substrate (IRS) proteins to resistance to PI3K inhibition. The insulin and insulin-like growth factor 1 (IGF1) receptors signal through IRS1 and IRS2 to activate PI3K/AKT signaling (31, 32, 33). Importantly, while both IRS1 and IRS2 activate PI3K and facilitate glucose homeostasis, IRS2 uniquely promotes breast cancer cell invasion and cancer stem cell self-renewal through ligand-dependent activation of PI3K signaling (34, 35). These functional differences raise the possibility that IRS1 and IRS2 may also have divergent contributions to PI3K pathway inhibitor sensitivity. Because of the importance of upstream PI3K signaling in mediating resistance to PI3K inhibition, and the role of IRS proteins in receptor-mediated PI3K signaling, we investigated the interplay among *PIK3CA* mutations, PI3K pathway inhibitors, and IRS proteins in breast cancer. We report that IRS2 expression confers resistance to PI3K pathway inhibition in *PIK3CA* mutant, but not *PIK3CA* WT breast cancer.

## Results

### PI3K signaling controls IRS2 mRNA and protein abundance in PIK3CA mutant breast cancer

**Figure 1:**
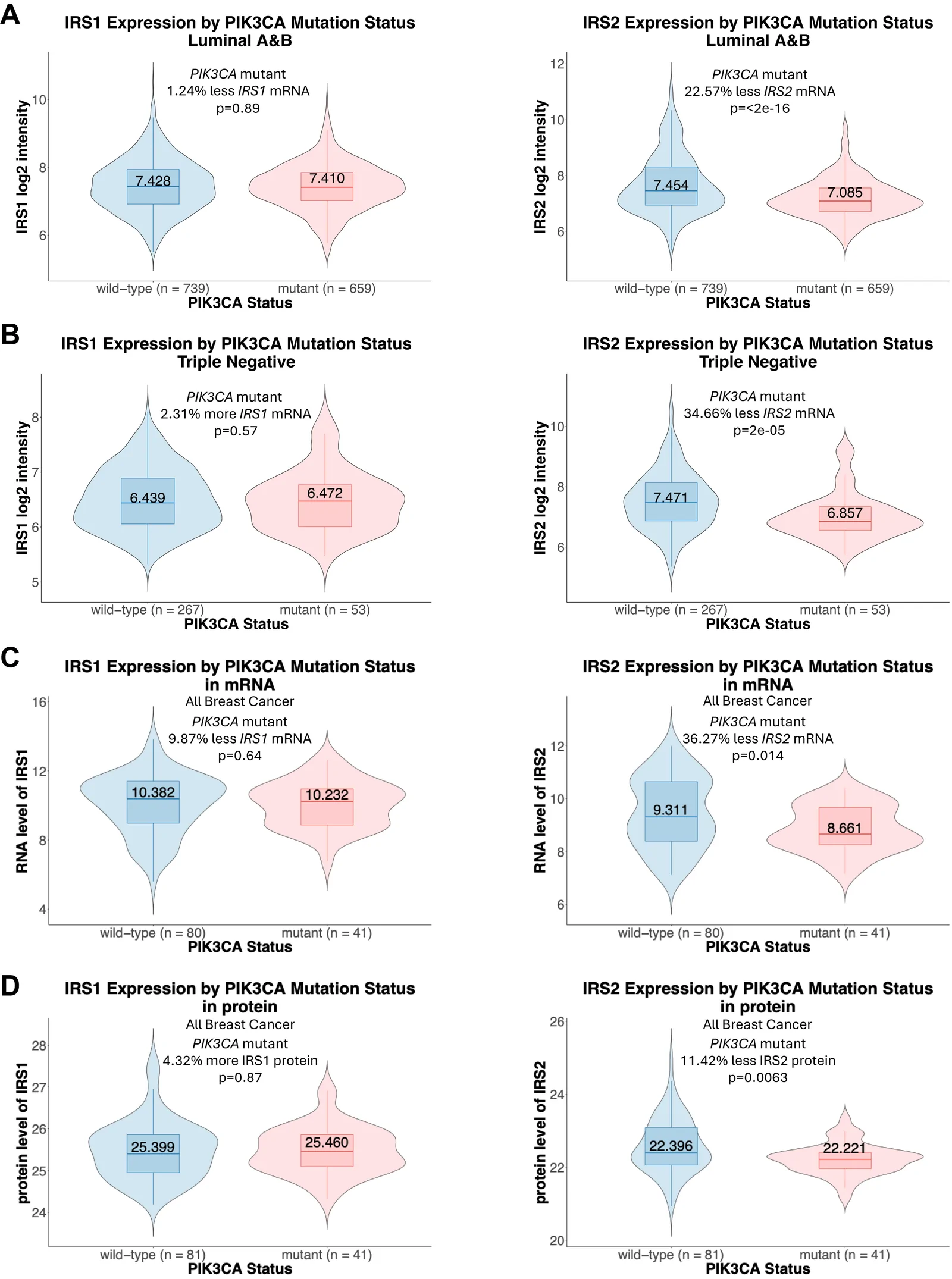
**Reduction of IRS2 mRNA and protein abundance in *PIK3CA* mutant breast cancer**. Differential gene expression analysis of IRS1 and IRS2 in *PIK3CA* WT and *PIK3CA* mutant luminal A&B (*A*) and triple-negative (*B*) breast tumors in the METABRIC dataset. Gene expression measurements shown as log2 intensity values (Illumina HT-12 v3 microarray). Differential mRNA (*C*) and protein (*D*) expression analysis of IRS1 and IRS2 in *PIK3CA* WT and *PIK3CA* mutant breast tumors in the Clinical Proteomic Tumor Analysis Consortium (CPTAC) dataset (comparisons include all breast cancer types). mRNA expression shown as Log2(RSEM+1), protein expression shown as Log2(MS1 intensity). For all violin plots, the box and whisker plots show the median, 25%(Q1), 75%(Q3) of data (box), Q1 - 1.5 × interquartile range (IQR), and Q3 + 1.5 × IQR of data (whiskers). For all panels, percent changes were determined by comparing the medians of the original data (prior to log transformation). For all panels, statistical analysis by Wilcoxon rank sum test with continuity correction. See also Figure S1. IRS, insulin receptor substrate.

To assess the impact of activating *PIK3CA* mutations on IRS adaptor proteins, we first examined whether *PIK3CA* mutations were associated with IRS1 or IRS2 abundance in breast cancer patient samples from the Molecular Taxonomy of Breast Cancer International Consortium dataset (36, 37). Whereas *IRS1* mRNA levels were similar in WT and mutant *PIK3CA* tumors, mutations in *PIK3CA* correlated with significant reductions in *IRS2* mRNA in luminal breast cancer, triple-negative breast cancer, and HER2-enriched breast cancer (Figs. 1, *A* and *B*, Fig. S1*A*) (36), findings consistent with previous reports (38, 39). This phenotype was also present in the independent Clinical Proteomic Tumor Analysis Consortium dataset, where *PIK3CA* mutations in breast cancer correlated significantly with reductions in both IRS2 mRNA and protein (Fig. 1, *C* and *D*) (40). Additional genomic alterations that lead to PI3K/AKT pathway activation, including *PTEN* deletion and *PTEN* mutation, also correlated with reduced *IRS2* mRNA in breast cancer patient samples (Fig. S1, *B*–*E*) (36).

**Figure 2:**
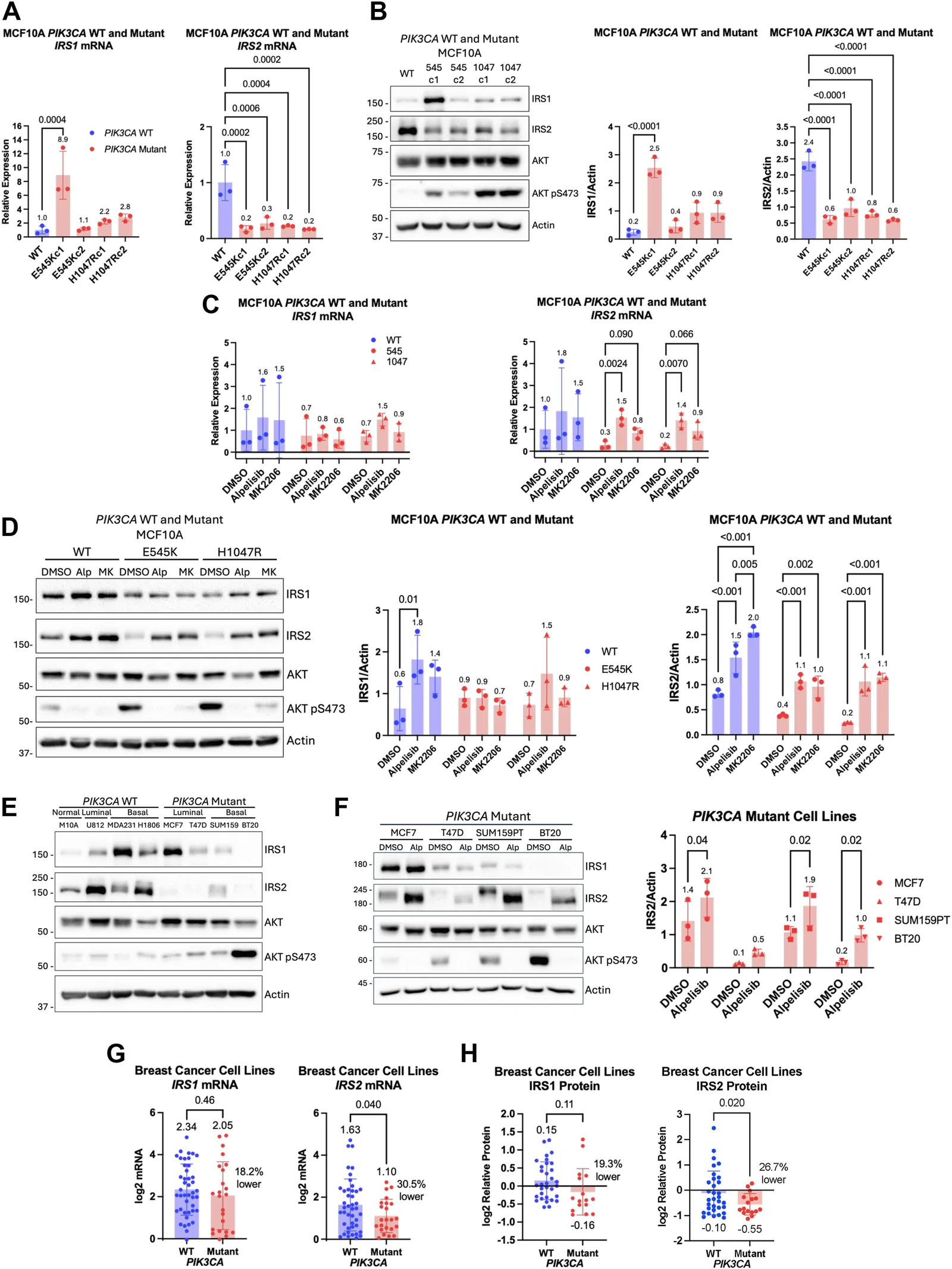
***PIK3CA* mutation reversibly suppresses IRS2 mRNA and protein abundance in mammary epithelial cells and breast cancer cell lines**. *A* and *B*, *PIK3CA* WT and *PIK3CA* mutant knockin MCF10A cells were serum starved, then evaluated for IRS1 and IRS2 mRNA (*A*) and protein (*B*) expression. The data shown in the graphs represent the mean ± standard deviation (SD) of three independent experiments. Statistical analysis by One-Way ANOVA followed by Dunnett’s multiple comparisons test to compare mutants against WT (*p* values shown). *C* and *D*, *PIK3CA* WT and *PIK3CA* mutant knockin MCF10A cells were treated in plain media with or without alpelisib (5 μM) or MK2206 (1 μM) for 24 h and then evaluated for IRS1 or IRS2 mRNA (*C*) or protein (*D*) expression. The data shown in the graphs represent the mean ± SD of three independent experiments. Statistical analysis by (*C*) separate one-way ANOVA followed by the Bonferroni test for multiple comparisons to compare drug treatments to DMSO or (*D*) Two-Way ANOVA followed by the Tukey test for multiple comparisons to compare all treatments within each cell type (*p* values shown). *E*, IRS1 and IRS2 protein expression in *PIK3CA* WT and *PIK3CA* mutant breast epithelial and cancer cell lines following serum starvation (M10A - MCF10A, U812 - UACC812, MDA-231 – MDA-MB-231, H1806 - HCC1806, SUM159 - SUM159PT). (*F*) *PIK3CA* mutant breast cancer cells were treated in plain media with or without alpelisib (5 μM) for 24 h and cell extracts were evaluated by immunoblot. The data shown in the graph represents the mean ± SD of three independent experiments. Statistical analysis by two-way ANOVA. Because these comparisons were planned *a priori*, no multiplicity adjustments were applied (*p* values shown). (*G* and *H*) Differential IRS1 and IRS2 mRNA (*G*) and protein (*H*) in breast cancer cell lines from DepMap. (mRNA - log2(TPM+1), protein RPPA signal). The data shown in the graphs represent the mean ± SD of all included cell lines. Percent changes were determined by comparing the means of the original data (prior to log transformation). Statistical analysis by Welch’s *t* test (two-tailed) (*p* values shown). See also Figure S2. IRS, insulin receptor substrate; DMSO, dimethyl sulfoxide.

Tumors often harbor multiple oncogenic mutations which make it difficult to attribute a given observation to a specific genomic alteration. To determine the isolated impact of activating *PIK3CA* mutations on IRS1 and IRS2 abundance, we utilized nontumorigenic MCF10A human mammary epithelial cells expressing *PIK3CA* WT or heterozygous knock-in of the two most common *PIK3CA* activating mutations, E545K or H1047R(2, 4). Importantly, these cells are isogenic, ensuring that any differential phenotypes are due to *PIK3CA* mutational status alone and not to other differences in mutational background. Two single-cell clones of each knock-in mutant were studied to control for clonal differences. *PIK3CA* mutant cells exhibited elevated PI3K/AKT signaling (Fig. S2*A*). WT and *PIK3CA* mutant cells expressed equivalent levels of IRS1 mRNA and protein in three of the four mutant clones, with one *PIK3CA* mutant clone expressing elevated IRS1 (Fig. 2, *A* and *B*). In contrast, all *PIK3CA* mutant clones expressed significantly lower IRS2 mRNA and protein levels compared to WT MCF10A cells (Fig. 2, *A* and *B*).

To assess if the reduction in IRS2 expression was related to increased PI3K signaling in *PIK3CA* mutant cells, *PIK3CA* WT and mutant cells were treated with alpelisib, a clinically approved PI3K p110α catalytic subunit specific inhibitor (hereafter referred to as PI3Kα) or an AKT1/2/3 inhibitor (MK2206). While both PI3Kα and AKT inhibition reduced AKT signaling as evidenced by lower AKT pS473 levels, neither PI3Kα nor AKT inhibition altered IRS1 mRNA or protein levels in *PIK3CA* mutant cells (Figs. 2, *C* and *D* and Fig. S2*B*). In contrast, both PI3Kα and AKT inhibition led to increased IRS2 mRNA and protein in WT as well as E545K and H1047R *PIK3CA* mutant cells (Figs. 2, *C* and *D* and Fig. S2*B*). This is consistent with a prior report of PI3K inhibition leading to increased *IRS2* mRNA in *PIK3CA* mutant cell lines (39).

While the use of MCF10A cells enabled us to assess the contribution of *PIK3CA* mutations to IRS1 and IRS2 abundance in cells lacking other oncogenic genomic alterations, they are not cancer cells. To determine if mutant *PIK3CA* also regulated IRS2 expression in breast cancer cell lines, IRS protein abundance was measured in three *PIK3CA* WT breast cancer cell lines and four *PIK3CA* mutant breast cancer cell lines. IRS1 expression was variable across all cell lines, but IRS2 expression was markedly reduced in all four *PIK3CA* mutant cell lines compared to *PIK3CA* WT cells (Fig. 2*E*). In each *PIK3CA* mutant cell line, PI3Kα inhibition yielded elevated IRS2 protein levels compared to vehicle treated controls (Fig. 2*F*). In contrast, PI3Kα inhibition in *PIK3CA* WT cells did not consistently alter IRS2 protein abundance (Fig. S2*C*). To assess if this selective *PIK3CA* phenotype was present in a larger number of breast cancer cell lines, we analyzed publicly available data from the Cancer Dependency Map (DepMap) (41). In these data, *PIK3CA* mutations were also associated with significantly reduced IRS2 mRNA and protein abundance, but IRS1 expression showed no association (Fig. 2, *G* and *H*).

### Mutant PIK3CA alters IRS2 expression through FOXO1/3

The strong influence of *PIK3CA* mutations and PI3K pathway inhibitors on *IRS2* mRNA abundance led us to hypothesize that active PI3K signaling regulates IRS2 expression through transcription factor regulation. While other IRS2 transcription factors have been described, we focused on FOXO1 and FOXO3 as they are regulated by PI3K/AKT signaling and do not drive IRS1 expression (42, 43). FOXO3 has also been previously implicated as playing a predominant role in the feedback regulation of IRS2 expression (42, 44, 45, 46, 47). PI3K signaling and *PIK3CA* mutations activate AKT, which phosphorylates FOXO1/3 in the nucleus, leading to FOXO1/3 nuclear exclusion and interrupting the ability of FOXO1/3 to facilitate target gene expression (22, 42, 43, 48, 49, 50, 51).

**Figure 3:**
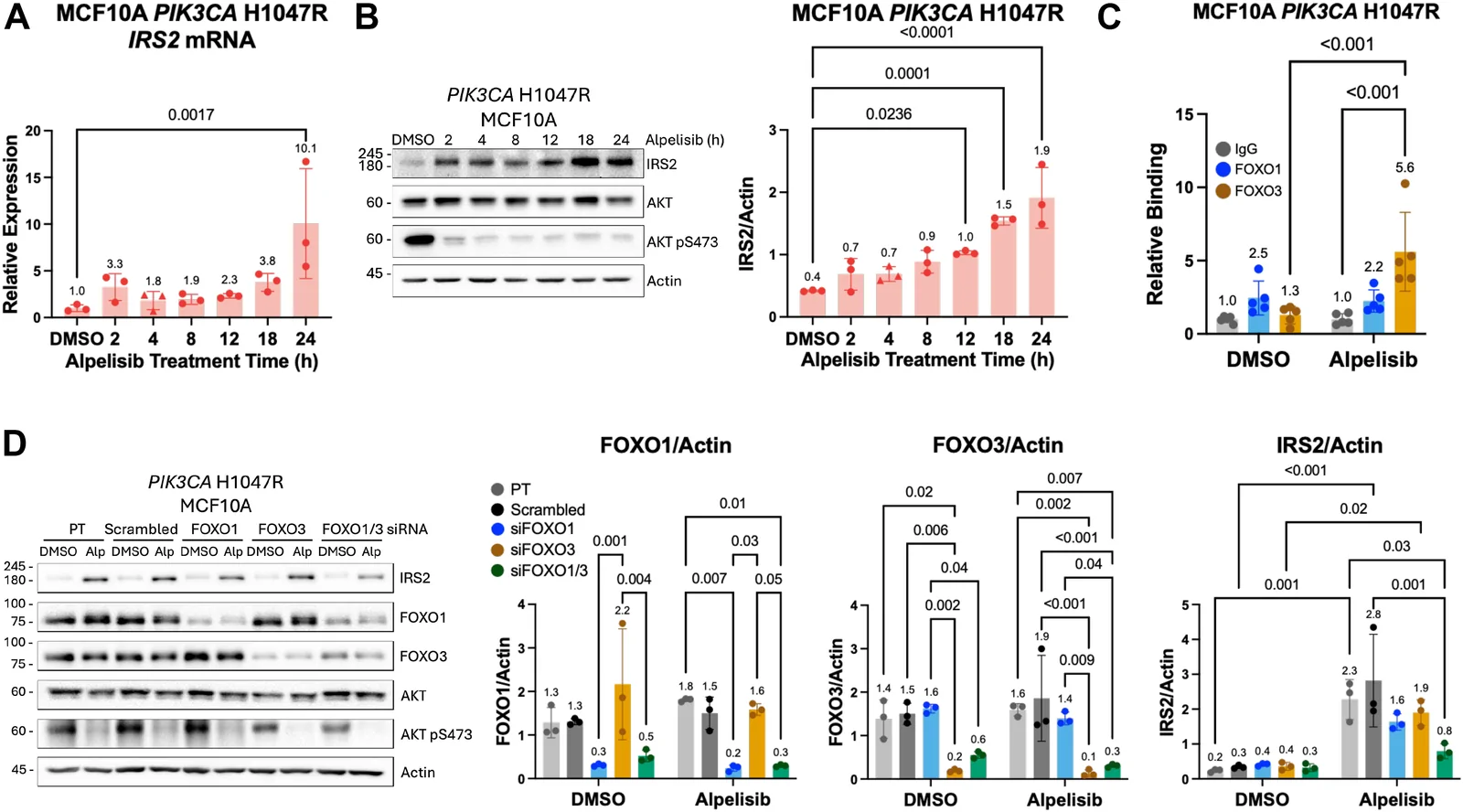
**Mutant *PIK3CA* regulates IRS2 expression through FOXO1 and FOXO3**. *A* and *B*, *PIK3CA* mutant H1047R MCF10A cells were treated with or without alpelisib (5 μM) for the time course indicated and then evaluated for IRS2 mRNA (*A*) and protein (*B*) expression. Cells were treated with DMSO for 24 h. The data shown represent the mean ± SD of three independent experiments. Statistical analysis by one-way ANOVA followed by Dunnett’s multiple comparisons test to compare DMSO against alpelisib treatments (*p* values shown). *C*, *PIK3CA* mutant H1047R MCF10A cells were treated in plain media with DMSO or alpelisib (5 μM) for 24 h. FOXO1 and FOXO3 binding to the IRS2 promoter was evaluated by ChIP-qPCR. The data shown are representative of independent experiments. Statistical analysis by two-way ANOVA. Because these comparisons were planned *a priori*, no multiplicity adjustments were applied (*p* values shown). *D*, *PIK3CA* mutant H1047R MCF10A cells were treated with siRNA for 72 h and DMSO or alpelisib (5 μM) were included for the final 24 h. Cell extracts were evaluated by immunoblot. The data shown in the graphs represent the mean ± SD of three independent experiments. Statistical analysis by two-way ANOVA followed by the Bonferroni test for multiple comparisons (*p* values shown). See also Figure S3. IRS, insulin receptor substrate; FOXO, Forkhead box protein O.

A time course treatment of cells with alpelisib revealed that IRS2 mRNA and protein expression increase significantly 18 to 24 h after inhibition of PI3Kα, a timing that reflects the nuclear translocation and transcriptional activity of FOXO family members (Fig. 3, *A* and *B*). PI3Kα inhibition in *PIK3CA* H1047R MCF10A cells led to increased nuclear FOXO1 and FOXO3. IRS2 nuclear localization also increased, which we have previously reported to be regulated in a PI3K-dependent manner (Fig. S3) (52). To examine the direct regulation of IRS2 by FOXO1/3, chromatin immunoprecipitation (ChIP)-quantitative polymerase chain reaction (qPCR) was performed using antibodies that recognize either FOXO1 or FOXO3 in the absence or presence of alpelisib. Although FOXO1 was present at low levels on the IRS2 promoter, only FOXO3 showed significantly increased binding in cells treated with alpelisib (Fig. 3*C*).

FOXO1/3-targeted siRNAs were utilized to suppress expression of these transcription factors individually and in combination to evaluate their functional role in mediating increased IRS2 expression in response to PI3Kα inhibition in *PIK3CA* mutant cells. Although a modest reduction was observed upon treatment with either FOXO1 or FOXO3 siRNAs alone, a combined suppression of both FOXO1 and FOXO3 was necessary to achieve a significant decrease in the induction of IRS2 expression in response to alpelisib-mediated inhibition of PI3Kα (Fig. 3*D*). Importantly, FOXO1 and FOXO3 expression levels were not altered in FOXO3 and FOXO1 KD cells, respectively. Taken together with the ChIP assays, our data support that both FOXO1 and FOXO3 contribute to regulating IRS2 expression, but that FOXO3 may play a more important role in the induction of IRS2 expression in response to PI3K pathway inhibition.

### IRS2 confers resistance to PI3K pathway inhibitors in PIK3CA mutant breast cancer

**Figure 4:**
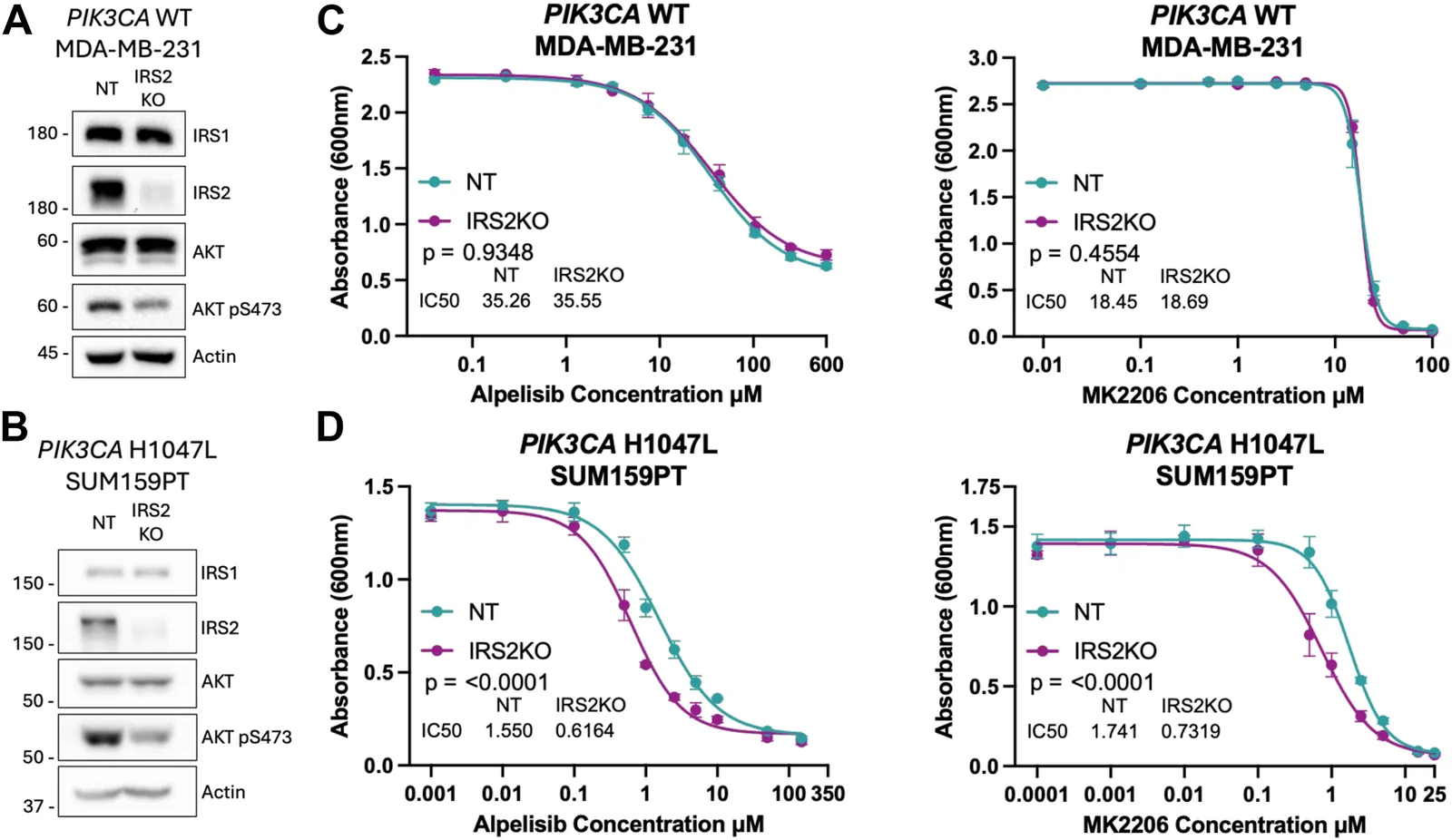
**IRS2 promotes resistance to PI3K pathway inhibitors in *PIK3CA* mutant breast cancer cells**. (*A*) MDA-MB-231 cells and (*B*) SUM159PT cells were treated with non-targeting guide RNA (*NT*) or IRS2-targeting guide RNA (IRS2KO) and cell extracts were analyzed by immunoblot. *C*, Viability (live cell population size) of *PIK3CA* WT MDA-MB-231 NT and IRS2KO cells treated with increasing concentrations of alpelisib or MK2206 for 72 h, measured by crystal violet staining. *D*, Viability of *PIK3CA* mutant SUM159PT NT and IRS2KO cells treated with increasing concentrations of alpelisib or MK2206 for 72 h, measured by crystal violet staining. *C* and *D*, statistical analysis by variable slope (four parameters) nonlinear regression using least squares regression to fit the curve (with asymmetrical confidence intervals calculated for parameters) and the extra sum-of-squares F test to compare IC50 values. Data shown are representative of three independent experiments. See also Figure S4.

IRS2 is an adaptor protein that links upstream receptor activation with PI3K/AKT signaling. While *PIK3CA* mutations increase baseline PI3K signaling, ligand stimulation can further augment PI3K activation, which is important given the role of sustained PI3K signaling in resistance to PI3K inhibitors (Fig. S4, *A* and *B*). To assess the role of IRS2 in resistance to PI3K pathway inhibition, we first generated control (NT) and IRS2-null (IRS2KO) MDA-MB-231 (*PIK3CA* WT) and SUM159PT (*PIK3CA* H1047L) cell lines using CRISPR-Cas9 gene editing (Fig. 4, *A* and *B*). Cells were plated, treated with increasing doses of PI3K pathway inhibitors for 72 h, fixed, and stained with crystal violet to measure the size of the cell population following drug treatment. Half-maximal inhibitory concentrations (IC50s) were estimated and used to compare drug sensitivity in different experimental conditions. Variable slope nonlinear regression was used to determine a best fit curve for the data and to estimate IC50 values (53). When *PIK3CA* WT cells were treated with alpelisib, control and IRS2KO cells exhibited equivalent drug sensitivity (Fig. 4*C*). This was also true when *PIK3CA* WT cells were treated with the AKT1/2/3 inhibitor MK2206 (Fig. 4*C*). In marked contrast, *PIK3CA* mutant (H1047L) cells lacking IRS2 exhibited increased sensitivity to PI3Kα and AKT inhibition compared to control cells, with IRS2KO IC50 values less than half of control IC50 values for alpelisib and MK2206 (Fig. 4*D*). A similar increased sensitivity of IRS2KO cells was observed when a dose response siRNA knockdown of PI3Kα was performed as a control for alpelisib drug specificity (Fig. S4, *C* and *D*).

To examine further if IRS2-dependent resistance to PI3K pathway inhibition is dependent upon *PIK3CA* mutation alone, experiments were repeated in isogenic MCF10A human mammary epithelial cells with WT *PIK3CA* or heterozygous knock-in of *PIK3CA* E545K or H1047R mutations (4). Control (NT) and IRS2KO *PIK3CA* WT, E545K, and H1047R cells were generated using CRISPR-Cas9 gene editing (Fig. S5*A*). For each cell line, two IRS2KO single-cell clones were isolated, maintained separately, and pooled together while plating cells for each viability experiment.

Crystal violet staining cannot determine if differences in cell proliferation or cell death are responsible for variations in cell population size. To determine if the increased sensitivity to PI3K pathway inhibition of *PIK3CA* mutant cells was caused by differences in cell proliferation or cell death, we conducted fluorescence-based and lysis-dependent inference of cell death kinetics (FLICK) assay experiments (53). Cells were incubated with increasing doses of drug and a fixed concentration of SYTOX Green that allows for the determination of the size of the live and dead cell populations at measured timepoints (53). These data can be used to calculate relative viability (RV), which is the size of the live cell population relative to the lowest drug dose, and is influenced by both cell proliferation and cell death (54, 55). FLICK can also be used to calculate Lethal Fraction (LF), which is the percent of cells that are dead. Together, these metrics characterize the cytostatic and cytotoxic effects of a drug. A decrease in RV may stem from decreased cell proliferation or increased cell death, or both, which illustrates the importance of measuring LF to determine how a drug is causing a decrease in cell population size.

**Figure 5:**
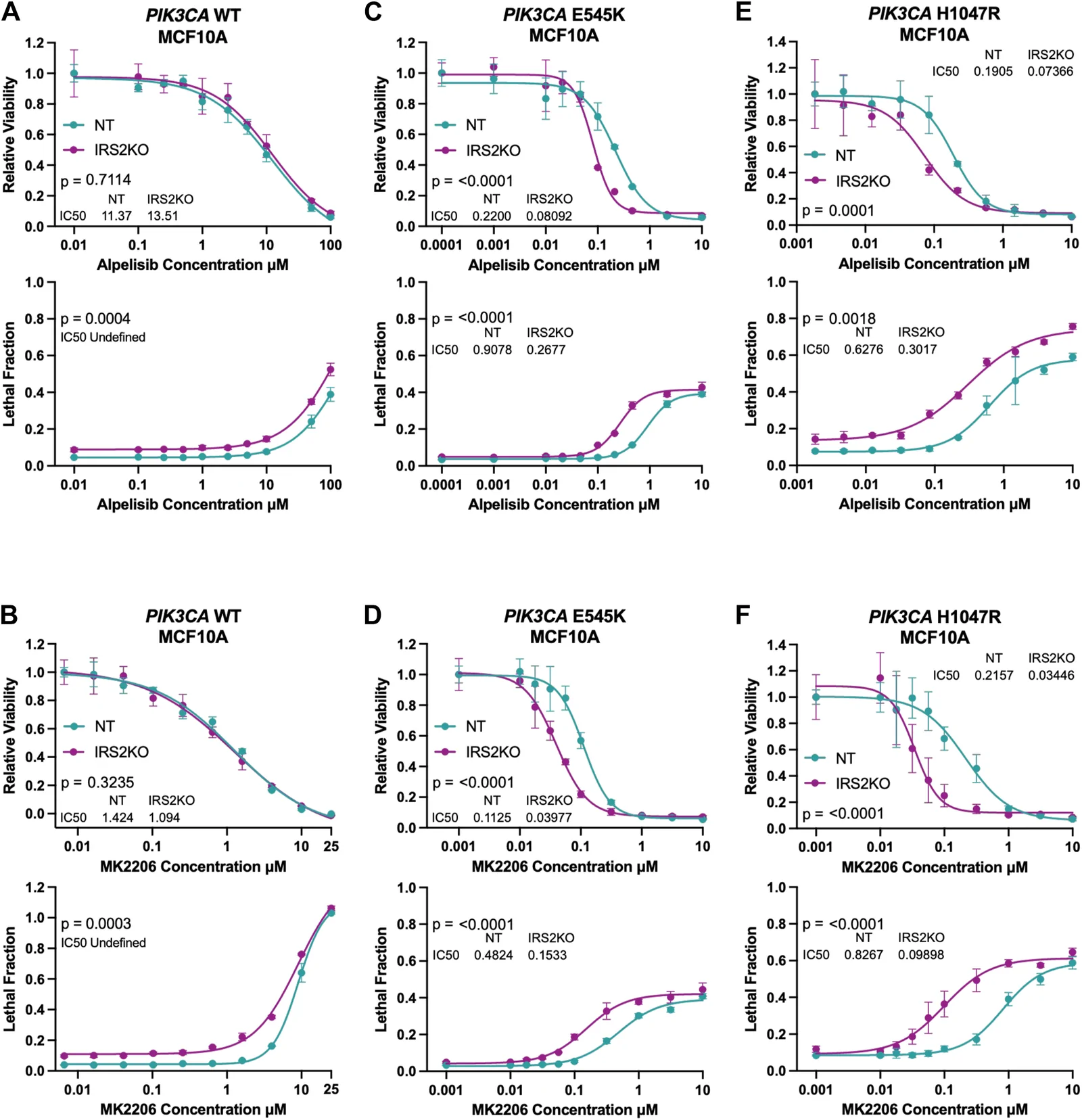
**IRS2 depletion reduces cell proliferation and increases cell death in *PIK3CA* mutant mammary epithelial cells in response to PI3K pathway inhibitors**. *A*, *C*, and *E*, Relative Viability (*top graph*) and Lethal Fraction (*bottom graph*) of NT and IRS2KO *PIK3CA* WT (*A*), *PIK3CA* E545K (*C*), and *PIK3CA* H1047R (*E*) MCF10A cells treated with increasing concentrations of alpelisib for 72 h, measured by FLICK assay. *B*, *D*, and *F*, Relative Viability (*top*) and Lethal Fraction (*bottom*) of NT and IRS2KO *PIK3CA* WT (*B*), *PIK3CA* E545K (*D*), and *PIK3CA* H1047R (*F*) MCF10A cells treated with increasing concentrations of MK2206 for 72 h, measured by FLICK assay. Statistical analysis by variable slope (four parameters) nonlinear regression using least squares regression to fit the curve (with asymmetrical confidence intervals calculated for parameters) and the extra sum-of-squares F test to compare IC50 values for all panels except Lethal Fraction for (*A*) and (*B*). Statistical analysis for Lethal Fraction for (*A*) and (*B*) by measuring area under curve (AUC) (GraphPad Prism). AUCs were compared using Welch’s *t* test (two-tailed), utilizing AUC total area and standard error. For AUC comparison of Lethal Fraction for (*A*) and (*B*), *p* values shown are for comparison of data after normalization to lowest drug dose for each subline. *p* values for original data (shown in graphs) are *p* < 0.0001 (*A* and *B*). For all panels, data shown are representative of three independent experiments. See also Figure S5. IRS, insulin receptor substrate; PI3K, phosphatidylinositol-3 kinase.

Consistent with the observations for WT *PIK3CA* expressing MDA-MB-231 cells, there was no difference in relative live cell population size (RV) between control and IRS2KO *PIK3CA* WT MCF10A cells treated with PI3Kα or AKT inhibitors (Fig. 5, *A* and *B*). In *PIK3CA* WT MCF10A cells, these drugs did induce cell death at high doses, and there was significantly more cell death in IRS2KO cells compared to control cells, as determined through area under curve (AUC) comparison. AUC comparison was utilized because incomplete cell death curves (LF) prevented accurate calculation of IC50 values (see Experimental procedures) (56, 57) (https://www.graphpad.com/support/faq/area-under-dose-response-data). The observation that differences in cell death (LF) did not cause differences in the relative live cell population sizes (RV) indicates that the differences in cell death between control and IRS2KO cells were modest.

In contrast to *PIK3CA* WT cells, both *PIK3CA* E545K and H1047R cells lacking IRS2 were more sensitive to PI3Kα and AKT inhibition compared to control cells, with IRS2KO cells exhibiting both reduced relative cell population size (RV) and increased cell death (LF) (Fig. 5, *C*–*F*). For IRS2KO cells, RV and LF IC50 values were less than half of control IC50 values for both inhibitors, similar to the increased sensitivity observed in IRS2KO *PIK3CA* mutant SUM159PT (H1047L) cells (Fig. 4*D*). Importantly, in the MCF10A *PIK3CA* mutant cell lines treated with PI3Kα or AKT inhibitors, differential cell death only partly accounted for differences in relative viability (RV), indicating that ablation of IRS2 also reduced proliferation in these cells.

**Figure 6:**
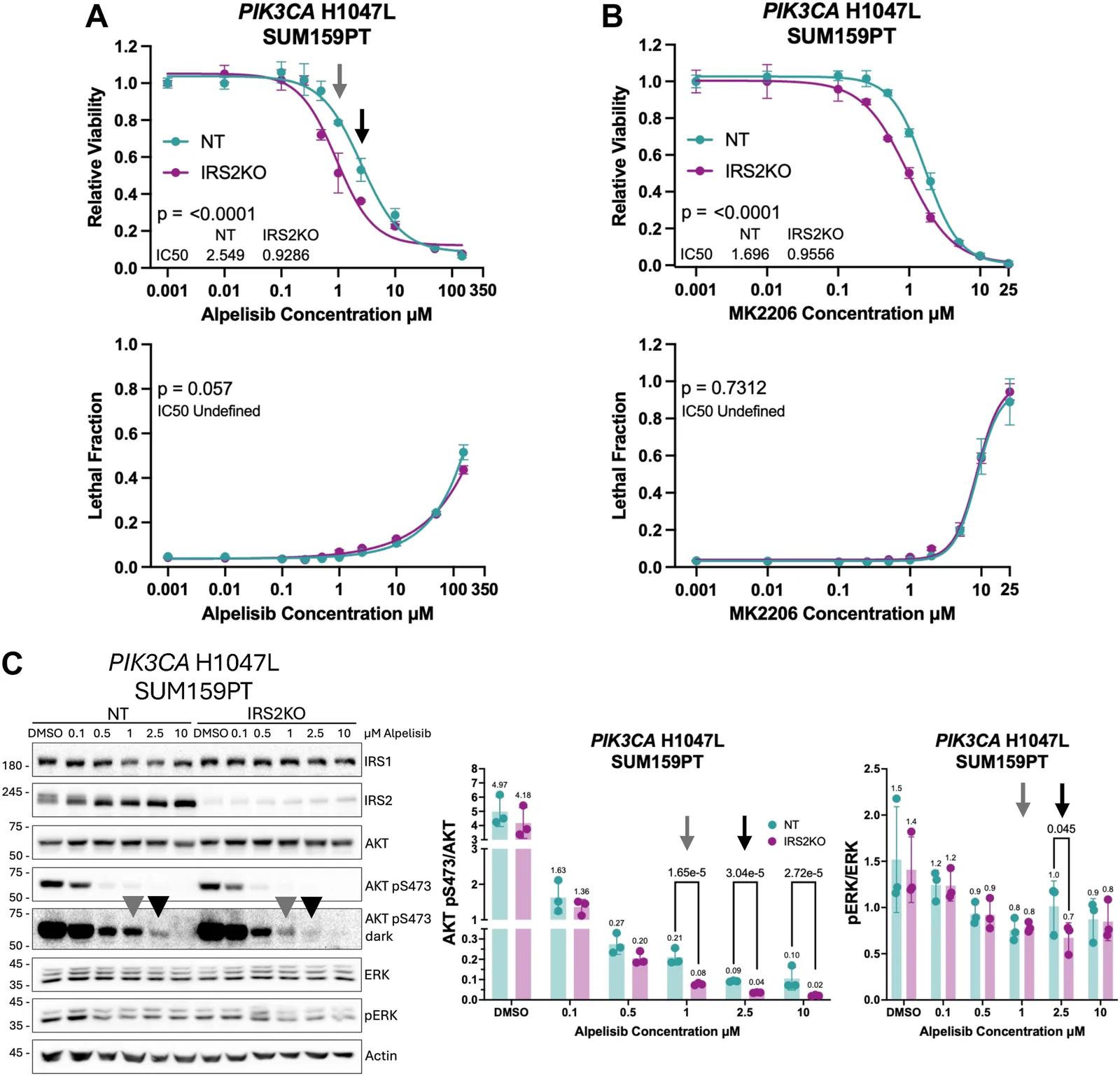
**IRS2 sustains PI3K signaling and cell proliferation in *PIK3CA* mutant breast cancer cells treated with PI3K pathway inhibitors**. *A* and *B*, Relative Viability (*top graph*) and Lethal Fraction (*bottom graph*) of NT and IRS2KO *PIK3CA* H1047L mutant SUM159PT cells treated with increasing concentrations of alpelisib (*A*) or MK2206 (*B*) for 72 h, measured by FLICK assay. Statistical analysis by variable slope (four parameters) nonlinear regression using least squares regression to fit the curve (with asymmetrical confidence intervals calculated for parameters) and the extra sum-of-squares F test to compare IC50 values for Relative Viability (*A* and *B*). Statistical analysis for Lethal Fraction for (*A*) and (*B*) by measuring area under curve (AUC) (GraphPad Prism). AUCs were compared using Welch’s *t* test (two-tailed), utilizing AUC total area and standard error. Data shown are representative of three independent experiments. (*C*) NT and IRS2KO *PIK3CA* H1047L mutant SUM159PT cells were treated with increasing concentrations of alpelisib for 24 h and cell extracts were evaluated by immunoblot. The data shown in the graphs represent the mean ± SD of three independent experiments. Statistical analysis was performed using a natural-log transformation followed by ordinary least squares (OLS) ANOVA on log(ratio), with fixed effects for genotype (NT *versus* IRS2KO), dose (categorical: 0 (DMSO), 0.1, 0.5, 1, 2.5, 10), their interaction, and a gel blocking factor, according to the model: log(ratio) ∼ genotype × dose + gel. Prespecified comparisons between NT and IRS2KO were conducted within each dose on the log scale. Because these comparisons were planned *a priori*, no multiplicity adjustments were applied (*p* values shown). IRS, insulin receptor substrate; PI3K, phosphatidylinositol-3 kinase; IRS2KO, IRS2-null.

To determine the impact of IRS2 depletion on cell proliferation and cell death in *PIK3CA* mutant tumor cells treated with PI3K pathway inhibitors, FLICK experiments were conducted using SUM159PT (*PIK3CA* H1047L) IRS2-WT (NT) and IRS2KO breast cancer cells. In these cells, both PI3Kα and AKT inhibition decreased the relative live cell population size (RV) in IRS2KO cells compared to control cells, with IRS2KO IC50 values significantly lower than control IC50 values for both alpelisib and MK2206 (Fig. 6, *A* and *B*). These results are consistent with findings in *PIK3CA* mutant MCF10A cells (Fig. 5), and with findings from crystal violet viability experiments in SUM159PT cells (*PIK3CA* H1047L) (Fig. 4). Interestingly, while both PI3Kα and AKT inhibition did induce cell death in the *PIK3CA* mutant cells at high doses (increase in LF), there was no difference in the percentage of cells that were alive between control and IRS2KO cells. This result contrasts with the results of MCF10A (*PIK3CA* E545K, H1047R) experiments (Fig. 5), in which IRS2KO cells exhibited differences in RV and in cell death (LF). This lack of increased cell death in IRS2KO SUM159PT cells reveals that the survival outcome of combined PI3K inhibition and IRS2 suppression is cell-context dependent. However, experiments across multiple *PIK3CA* mutant cell lines consistently show that IRS2KO cells exhibit increased sensitivity to PI3K pathway inhibition which leads to a reduction in cell proliferation.

### IRS2 promotes resistance to PI3K inhibition by activating PI3K signaling

To explore the potential mechanism(s) for IRS2-dependent resistance to PI3K pathway inhibition, we compared cell viability and IRS2-dependent signaling pathway activity during PI3Kα inhibition (Fig. 6*C*). PI3K/AKT and ERK signaling were assessed because IRS2 is known to activate both pathways and both pathways can regulate cell proliferation (58). Compared to control cells, there was a significant reduction in PI3K/AKT signaling (AKT pS473) in IRS2KO cells at doses of alpelisib (1 μM, 2.5 μM) that elicited divergent cell viability (Fig. 6, *A* and *C*). There was also reduced ERK signaling in IRS2KO cells as measured by ERK phosphorylation at 2.5 μM, although the difference between IRS2KO and control cells was smaller than for PI3K signaling.

**Figure 7:**
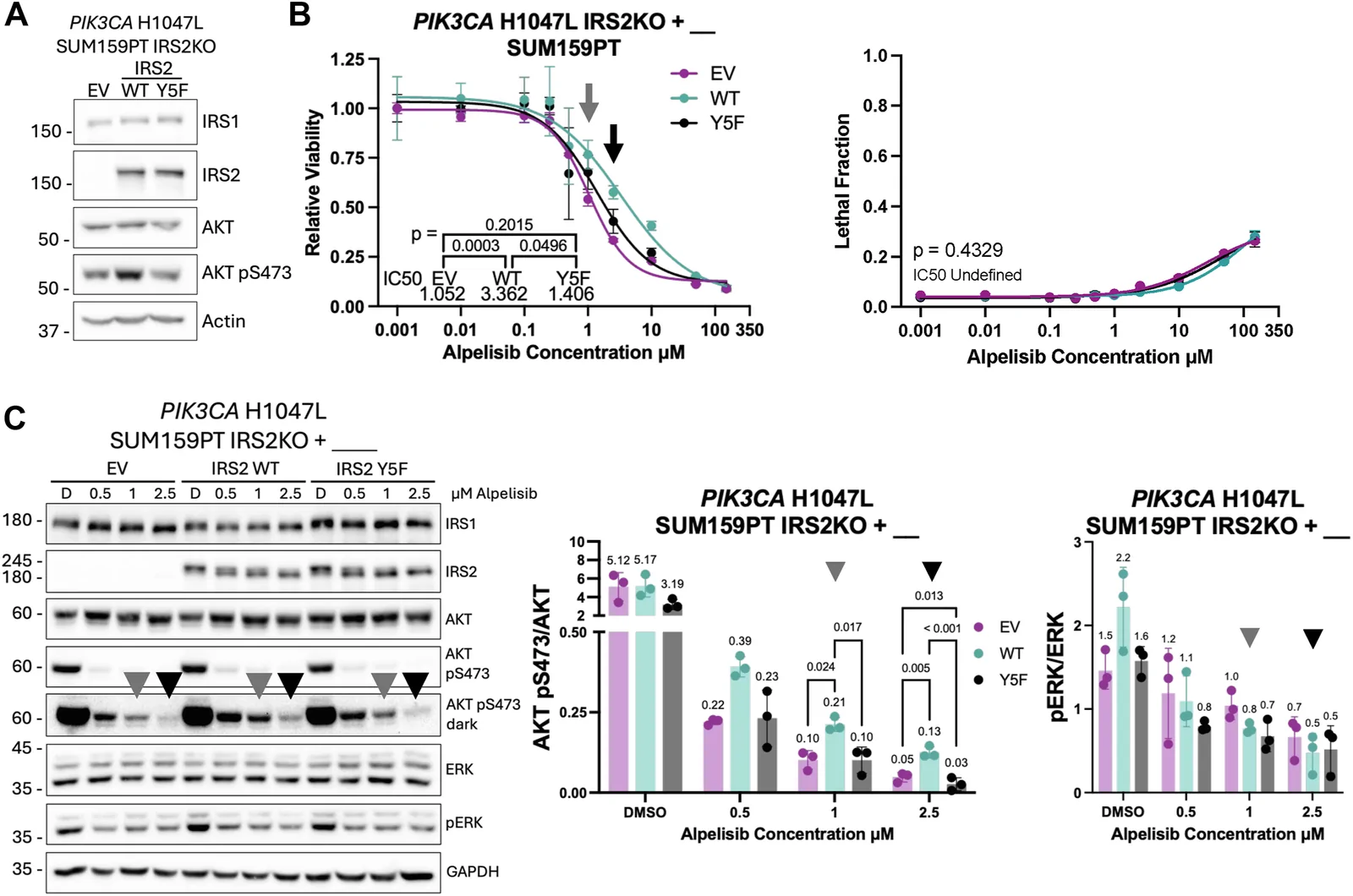
**IRS2-dependent PI3K activation confers resistance to PI3K inhibition in *PIK3CA* mutant breast cancer**. *A*, cell extracts from *PIK3CA* H1047L mutant SUM159PT IRS2KO cells expressing empty vector (EV), IRS2-WT or IRS2-Y5F were analyzed by immunoblot. *B*, Relative Viability (*left graph*) and Lethal Fraction (*right graph*) of *PIK3CA* H1047L mutant SUM159PT IRS2KO cells expressing empty vector (EV), IRS2-WT or IRS2-Y5F treated with increasing concentrations of alpelisib for 72 h, measured by FLICK assay. Statistical analysis of Relative Viability by variable slope (four parameters) nonlinear regression using least squares regression to fit the curve (with asymmetrical confidence intervals calculated for parameters) and the extra sum-of-squares F test to compare pairs of IC50 values. Statistical analysis for Lethal Fraction by measuring area under curve (AUC) (GraphPad Prism). AUCs were compared using One-Way ANOVA followed by the Tukey test for multiple comparisons to compare all treatments, utilizing AUC total area and standard error. Data shown are representative of three independent experiments. *C*, *PIK3CA* H1047L mutant SUM159PT IRS2KO cells expressing empty vector (EV), IRS2-WT or IRS2-Y5F were treated with increasing concentrations of alpelisib for 24 h and cell extracts were analyzed by immunoblot (D = DMSO). The data shown in the graphs represent the mean ± SD of three independent experiments. Statistical analysis was performed using a natural-log transformation followed by ordinary least squares (OLS) ANOVA on log(ratio), with fixed effects for genotype (EV *versus* IRS2-WT *versus* IRS2-Y5F), dose (categorical: 0 (DMSO), 0.5, 1, 2.5), their interaction, and a gel blocking factor, according to the model: log(ratio) ∼ genotype × dose + gel. Prespecified comparisons between each genotype pair within each dose were conducted on the log scale. Because these comparisons were planned *a priori*, no multiplicity adjustments were applied (*p* values shown). See also Figure S6. IRS, insulin receptor substrate; PI3K, phosphatidylinositol-3 kinase; DMSO, dimethyl sulfoxide.

To further probe the mechanism of IRS2-mediated resistance to PI3K pathway inhibition, IRS2KO SUM159PT cells (*PIK3CA* H1047L) were transduced to express empty vector (EV) (control), WT IRS2 (IRS2-WT), or IRS2-Y5F, which is a full-length mutant form of IRS2 which includes five tyrosine to phenylalanine mutations that block IRS2 recruitment of the p85 subunit of PI3K (Fig. 7*A*) (59). IRS2 Y5F is useful because it only interrupts the ability of IRS2 to activate PI3K, it does not affect ERK signaling (60). Both IRS2 WT and IRS2 Y5F were expressed at equivalent levels (Figs. 7*A*, S6*A*). *PIK3CA* mutant cells rescued with IRS2-WT expression were more resistant to PI3Kα inhibition compared to EV control cells (Fig. 7*B*). However, cells expressing IRS2-Y5F retained a greater sensitivity to PI3Kα inhibition, similar to EV control cells. The magnitude of the EV and WT-IRS2 effect (3-fold lower IC50 values) was consistent with prior experiments in SUM159PT NT and IRS2KO cells (2.5-fold lower IC50 values) (Figs. 4*D* and 6*A*). Also consistent with these prior experiments, the restoration of IRS2-WT expression did not impact cell death.

At alpelisib doses that yielded differences in viability (1 μM, 2.5 μM) (Fig. 7*B*), cells expressing WT-IRS2 sustained PI3K signaling relative to EV control and IRS2-Y5F cells (Fig. 7*C*). In contrast, there were no differences in ERK activity between EV control, IRS2-WT, and IRS2-Y5F expressing cells. Although IRS1 is not upregulated by PI3K inhibition, it can also activate PI3K signaling. Overexpression of IRS1 in IRS2KO cells rescued PI3K activity and also restored alpelisib resistance (Fig. S6, *B* and *C*). The inability of IRS2-Y5F to restore resistance to PI3K inhibition, coupled with reductions in PI3K signaling which correspond with increased sensitivity to alpelisib, indicates that IRS2-dependent PI3K signaling mediates resistance to PI3K inhibition in *PIK3CA* mutant cells.

### IRS2 expression influences PI3K pathway inhibitor sensitivity across PIK3CA mutant cancers

**Figure 8:**
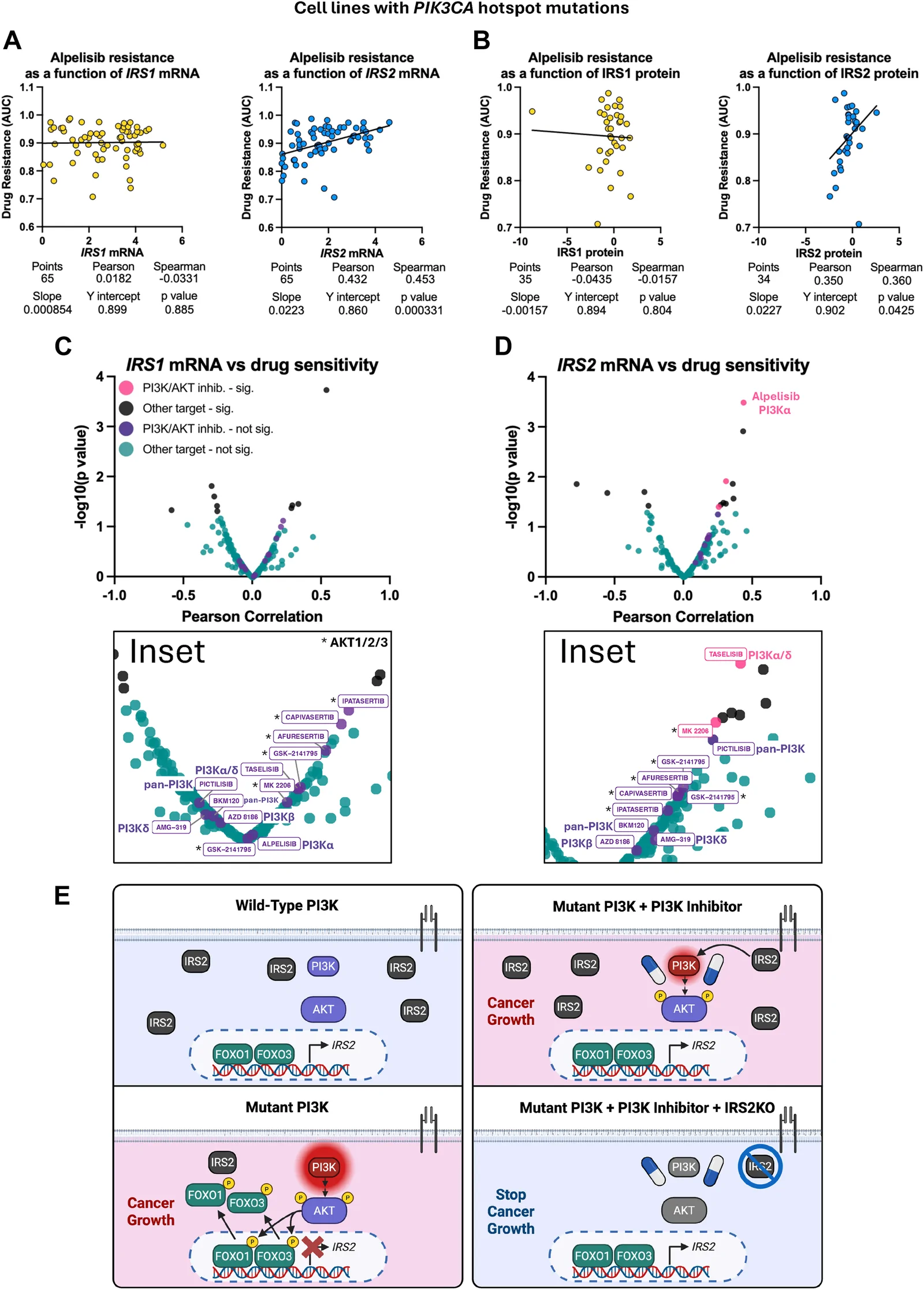
**IRS2 expression positively correlates with resistance to PI3K pathway inhibitors in *PIK3CA* mutant cancer cell lines**. *A* and *B*, correlation between IRS1 and IRS2 mRNA (*A*) or protein (*B*) and sensitivity to alpelisib in cancer cell lines with *PIK3CA* hotspot mutations (excluding myeloid and lymphoid-derived cell lines) in the DepMap dependency dataset. *p* values shown are from simple linear regression, *p* < 0.05 indicates that the slope is significantly different from zero. mRNA abundance shown is log2(TPM+1). *C* and *D*, correlation between *IRS1* (*C*) and *IRS2* (*D*) mRNA (DepMap) and sensitivity to drugs tested in the Sanger Genomics of Drug Sensitivity in Cancer 2 (GDSC2) drug panel in *PIK3CA* mutant cancer cell lines (excluding myeloid and lymphoid-derived cell lines) (accessed using the DepMap dependency dataset). Drug sensitivity was determined by AUC. GSK-2141795 was tested twice in GDSC2 and both results are plotted. Pearson correlation of mRNA abundance (log2(TPM+1)) and drug sensitivity (AUC). *p* values (y axis) are from simple linear regression of mRNA abundance *versus* drug sensitivity. Significance defined as *p* < 0.05. Abbreviations, Sig. for significant, inhib. for inhibitor. Drug names and targets listed (inset). Pan-PI3K – inhibitor of PI3Kα/β/δ/*γ*. *E*, model for IRS2 conferring resistance to PI3K pathway inhibition in PI3K-mutant breast cancer. *PIK3CA* mutations activate PI3K signaling to inactivate FOXO1 and FOXO3, leading to reduced IRS2 mRNA and protein abundance. PI3K inhibition leads to FOXO1 and FOXO3-driven increases in IRS2. During PI3K inhibition, IRS2 sustains PI3K signaling and promotes *PIK3CA* mutant cancer cell viability. Ablation of IRS2 during PI3K inhibition lowers PI3K signaling, leading to reduced cancer cell population growth (Created in BioRender. https://BioRender.com/zbf3joy). See also Figure S7. IRS, insulin receptor substrate; PI3K, phosphatidylinositol-3 kinase; FOXO, Forkhead box protein O.

To examine if IRS2 confers resistance to PI3K pathway inhibitors in a wider cancer context, the Cancer Dependency Map (DepMap) was used to generate correlations between IRS1 or IRS2 mRNA or protein abundance and PI3Kα inhibitor sensitivity (alpelisib) for all *PIK3CA* mutant cell lines arising from solid tumors (Fig. 8) (41). No relationship between IRS1 mRNA or protein levels and PI3Kα inhibitor sensitivity was observed (Fig. 8, *A* and *B*). However, *IRS2* mRNA abundance correlated with PI3Kα inhibitor sensitivity, with higher levels of *IRS2* associated with increased resistance to PI3Kα inhibition (as measured by increased AUC) (Fig. 8*A*). This correlation was also present for IRS2 protein levels (Fig. 8*B*). To interrogate if this association was unique to *PIK3CA* mutant cell lines, *IRS1* and *IRS2* mRNA levels were correlated to PI3Kα inhibitor sensitivity in *PIK3CA* WT cells arising from all solid tumors (Fig. S7*A*). *IRS1* did not correlate with drug sensitivity, whereas *IRS2* showed a modest positive and significant association with resistance to PI3Kα inhibition. However, linear regression on data from *PIK3CA* mutant cells exhibited a steeper slope and higher Pearson correlation coefficient compared to the *IRS2*-alpelisib sensitivity plot from *PIK3CA* WT cells, indicating that *IRS2* mRNA abundance had a larger impact on alpelisib sensitivity in *PIK3CA* mutant cells than in WT cells. A similar trend was observed for cell line sensitivity to the AKT inhibitor MK2206, with only *IRS2* being associated with drug sensitivity in *PIK3CA* mutant cells (Fig. S7*B*).

We expanded these analyses by assessing the correlation of *IRS1* and *IRS2* expression with sensitivity to a broad panel of cancer targeting drugs, that included additional agents targeting the PI3K pathway, in *PIK3CA* mutant cancer cell lines arising from solid tumors using DepMap (Fig. 8, *C* and *D*) (41, 61, 62). Higher *IRS2* expression significantly correlated with resistance to alpelisib and MK2206, which were both used in our studies (Fig. 8*D*). A significant association with resistance was also observed for taselisib, a PI3Kα/δ-selective inhibitor. The only PI3Kα-selective inhibitors included in the drug panel, alpelisib and taselisib, had the first and third highest correlation with *IRS2* expression of all compounds tested, respectively. This raises the possibility that IRS2 depletion would be most sensitizing to PI3Kα inhibitors, several of which have been approved clinically for the treatment of *PIK3CA* mutant breast cancer (https://www.fda.gov/drugs/resources-information-approved-drugs/fda-approves-alpelisib-metastatic-breast-cancer, https://www.fda.gov/drugs/resources-information-approved-drugs/fda-approves-inavolisib-palbociclib-and-fulvestrant-endocrine-resistant-pik3ca-mutated-hr-positive). In contrast, *IRS1* expression was not significantly associated with resistance to any of the PI3K or AKT inhibitors tested (Fig. 8*C*). While it will be important to functionally evaluate cell lines representative of other cancer types for their sensitivity to PI3K pathway inhibitors, these observations support that IRS2 may promote resistance to PI3K pathway inhibitors in *PIK3CA* mutant cancers from a variety of tissues.

## Discussion

In this study, we establish a compensatory feedback mechanism by which PI3K pathway inhibitors increase IRS2 abundance and demonstrate the role of IRS2 in promoting resistance to these drugs (Fig. 8*E*). IRS2 mRNA and protein levels are lower in *PIK3CA* mutant breast cancer patient tumors and cell culture models of breast cancer. This phenotype involves PI3K-dependent inactivation of the transcription factors FOXO1/3 and is reversed by PI3Kα or AKT inhibition. In contrast, IRS1 expression is not altered by *PIK3CA* mutations. IRS2 expression confers resistance to PI3K pathway inhibitors in *PIK3CA* mutant, but not *PIK3CA* WT breast cancer, by sustaining PI3K signaling. Increased IRS2 abundance correlates significantly with resistance to many PI3K pathway inhibitors across cancer cell lines arising from diverse tissues. Our findings have clinical relevance given the frequency of *PIK3CA* mutations in cancer, the importance of PI3K signaling reactivation in resistance to PI3K pathway inhibitors, and the identification of a new target to address the challenges associated with prior efforts to block PI3K pathway reactivation during PI3K inhibition.

Analysis of publicly available data revealed that IRS2 mRNA and protein abundance is decreased in *PIK3CA* mutant human breast tumors and breast cancer cell lines. This reduced level of IRS2 expression may seem counterintuitive given the previously demonstrated role of IRS2 in promoting tumor progression and metastasis and the more aggressive nature of *PIK3CA* mutant tumors. However, although IRS2 is expressed at lower levels in these cells, it remains functionally important. For example, in SUM159PT cells (*PIK3CA* H1047L), stimulation with insulin or IGF-1 further enhances PI3K pathway activity, and IRS2 is required for efficient invasion and breast cancer stem cell self-renewal (34, 35, 52). *PIK3CA* mutant cells may require lower levels of IRS2 because the majority of the necessary PI3K signaling is provided through mutant PI3K. However, when *PIK3CA* mutant cells experience reduced PI3K signaling due to PI3K pathway inhibition, compensatory pathways for PI3K activation are necessary and elevated levels of IRS2 are required to maintain adequate PI3K signaling for continued viability. The insulin-PI3K-FOXO regulatory feedback pathway that plays an important role in controlling normal metabolic homeostasis is highjacked in *PIK3CA* mutant tumor cells to sustain viability in response to targeted pathway inhibitors. One aspect of note from our study is that IRS2 ablation sensitizes *PIK3CA* mutant cells, but not *PIK3CA* WT cells, to PI3K pathway inhibitors. In line with this finding, our results, as well as prior literature, indicate that *PIK3CA* mutant cancer cells are more sensitive to PI3K pathway inhibitors compared to *PIK3CA* WT cancer cells (14, 63, 64, 65). This may be due to an increased dependence on the upregulation of compensatory PI3K signaling mechanisms, such as IRS2, during PI3K pathway inhibition in *PIK3CA* mutant cancer cells compared to *PIK3CA* WT cells. This also explains why a previous effort to identify mechanisms of resistance to PI3K inhibition that did not evaluate *PIK3CA* WT and mutant cells separately failed to identify IRS2 as a driver of PI3K inhibitor resistance, as IRS2 ablation sensitizes only *PIK3CA* mutant cells to PI3K pathway inhibition (66, 67, 68).

Our findings on the role of IRS2 in promoting PI3K pathway inhibitor resistance suggest that other molecules associated with IRS2 function and signaling could also be involved. For example, several groups have reported that PI3K pathway inhibitors cause IGF1R upregulation, which leads to reactivation of PI3K signaling and drug resistance (27, 28, 30). Other studies implicate MYC in resistance to PI3K pathway inhibitors (24, 69). Previous work from our lab reported that IGF1R signals through IRS2, but not IRS1, to stabilize MYC and support breast cancer stem cell activity, (35). IRS2-dependent MYC stabilization could also explain why IRS2 ablation coupled with PI3K pathway inhibition limits cell proliferation. Given the role of IRS2 in activating PI3K and AKT, which drive cell survival, and the previously described role of IRS2 in promoting cell survival, it was somewhat surprising that IRS2 loss coupled with PI3K pathway inhibition increased cell death in MCF10A *PIK3CA* mutant cells, but not in *PIK3CA* mutant SUM159PT cells (70). However, these knock-in MCF10A cells carry no mutations other than mutant *PIK3CA*, whereas SUM159PT cells harbor mutations in *PIK3CA* as well as *TP53* and *HRAS*, which may compensate for the loss of IRS2 and promote cell survival in its absence (71). Analysis of a broader number of *PIK3CA* mutant tumor cell lines will be necessary to determine the factors that influence cytostatic *versus* cell death responses when IRS2 expression is suppressed.

Although our studies were performed using cell lines, our analysis of publicly available breast cancer tumor datasets revealed a correlation between IRS2 expression and *PIK3CA* mutations across all breast cancer subtypes. PI3K pathway mutations occur frequently in HR+ breast cancer and activation of the PI3K signaling pathway is a known mediator of endocrine therapy resistance in HR+ breast cancer (2, 14, 72, 73). Combining PI3K inhibitors with endocrine therapy has shown improvement in breast cancer patient response rates, however adverse metabolic toxicities remain a problem and can be a limiting factor in this combination therapy (14, 15). Our findings raise the possibility that HR+ patients could also benefit from a combination of endocrine therapy, PI3Kα inhibition, and IRS2 suppression that would both limit metabolic toxicities and inhibit tumor growth.

From a clinical perspective, the frequency and severity of adverse events caused by PI3K pathway inhibitors limits their utility. In a phase 3 clinical trial of alpelisib, the PI3Kα specific inhibitor used in our study, over 60% of patients had to take a reduced dose due to side effects, and 25% of patients had to stop the treatment altogether (14). Other therapies targeting this pathway also suffer from frequent dose-limiting side effects (15, 74). Treatment-induced hyperglycemia and gastrointestinal symptoms, which constitute some of the most frequent severe side effects, stem from inhibition of PI3K in nontumor tissues (75). One approach to reducing side effects during PI3K inhibition is for patients to limit hyperglycemia by adhering to a low carbohydrate, ketogenic diet. While this approach attenuates PI3K-inhibitor induced hyperglycemia and hyperinsulinemia in mouse models of cancer and is being tested clinically, it is not a perfect solution, as patients often have difficulty adhering to a strict ketogenic diet (19, 76) (https://clinicaltrials.gov/study/NCT05090358). Use of an SGLT2 inhibitor to decrease hyperglycemia during PI3K inhibition is also being evaluated (https://clinicaltrials.gov/study/NCT05090358). While carbohydrate restriction and SGLT2 inhibition may minimize hyperglycemia in patients, these approaches are unlikely to reduce gastrointestinal side effects because these side effects are due to direct PI3K inhibition in these tissues, not hyperglycemia.

Sensitizing *PIK3CA* mutant cancers to PI3K pathway inhibitors through depletion of IRS2 could be a promising approach to improve clinical outcomes because patients would require lower drug doses (Fig. 8*E*). Targeting IRS2 would also interfere with feedback insulin signaling that drives tumor growth. This would limit all on-target and off-target side effects, rather than only those due to hyperglycemia, while allowing IRS1 to signal normally for metabolic regulation. This metabolic redundancy may lead to a better safety profile for IRS2 depletion coupled with PI3K inhibition compared to blocking PI3K pathway reactivation during PI3K inhibition by targeting IGF1R or mTOR, as side effects from these combinations limit drug tolerability (https://clinicaltrials.gov/study/NCT01708161) (77, 78, 79). IRS2 depletion decreased alpelisib IC50 values by 2.5-fold across several *PIK3CA* mutant cell lines. A dose escalation study found that patients receiving a 50% reduction in alpelisib daily (300 mg *versus* 150 mg) were 3 times less likely to experience severe hyperglycemia (80). This finding shows the dose dependent nature of alpelisib-induced side effects and highlights the opportunities for combination therapies, such as IRS2 depletion, to sensitize breast cancer to PI3K pathway inhibition, allowing lower drug dosages that minimize adverse events.

PI3K inhibitors are largely cytostatic when given as a monotherapy. Combination therapy that includes targeting IRS2 would likely enhance the cytostatic effects of PI3K inhibition in *PIK3CA* mutant cells (as seen in SUM159PT cells) and may induce cell death in specific mutational backgrounds (as seen in MCF10A *PIK3CA* mutant cells). Moreover, combining IRS2 suppression with lower alpelisib doses could sustain cytostatic tumor effects while also improving metabolic toxicities and allowing patients to continue treatment. IRS2 has received limited attention as a potential therapeutic target because it is a cytoplasmic adaptor protein with no active site and is therefore not amenable to inhibition with antibodies or small molecules, respectively. While one IRS-targeting agent has been reported, it has pleiotropic effects that suppress both IRS1 and IRS2 indirectly and has not been pursued clinically (81, 82). We recently demonstrated that siRNAs conjugated to an albumin binding dendrimer selectively suppress *Irs2*, but not *Irs1*, expression in mammary tumors without causing hyperglycemia (83). With the emergence of these RNA-based gene silencing drug modalities, depletion of IRS2 in combination with a PI3K/AKT inhibitor may be a feasible future approach for the treatment of *PIK3CA* mutant cancer.

## Experimental procedures

### Cells and cell culture

MCF10A cells (*PIK3CA* WT and *PIK3CA* knock-in mutant cells) were a gift from Dr Ben Ho Park (Vanderbilt) and were authenticated by STR profiling at the University of Arizona Genetics Core. *PIK3CA* mutant MCF10A cells were maintained in DMEM/F12 (Gibco) containing 5% horse serum (Gibco), 0.5 μg/ml hydrocortisone (Sigma-Aldrich), 100 ng/ml cholera toxin (Sigma-Aldrich), 10 μg/ml insulin (Sigma-Aldrich), and 100U/ml Penicillin-Streptomycin (Gibco). *PIK3CA* WT MCF10A cells were maintained in identical media that also contained 20 ng/ml human EGF (Sigma-Aldrich). *PIK3CA* WT and mutant MCF10A cells were plated in the same media for 18 to 24 h prior to starting experiments to normalize the experimental conditions. Importantly, differences in IRS2 expression between the WT and *PIK3CA* mutant cell lines in their normal culture conditions, as well as the induction of IRS2 expression in response to PI3Kα inhibition, were maintained after this preincubation period in the common medium. SUM159PT cells were obtained from BioIVT and were authenticated by STR profiling at the University of Arizona Genetics Core. They were grown in F12 media (Gibco) containing 5% FBS (Cytiva), 25 mM Hepes (Sigma-Aldrich), 5 μg/ml insulin (Sigma-Aldrich) and 1 μg/ml hydrocortisone (Sigma-Aldrich). MDA-MB-231, T47D, and MCF7 human breast carcinoma cells were obtained from the ATCC Cell Biology Collection. MDA-MB-231 and T47D cells were grown in RPMI (Gibco) containing 10% FBS (Cytiva). MCF7 cells were grown in MEM (Gibco) containing 10% FBS (Cytiva) and 11.2 μg/ml insulin (Sigma-Aldrich). UACC812 and HCC1806 human breast carcinoma cells were a gift from Dr Dohoon Kim (UMass Chan). UACC812 cells were maintained in DMEM 4.5 g/L glucose (Gibco) containing 100U/ml Penicillin-Streptomycin (Gibco), 2 mM L-Glutamine (Gibco), and 10% FBS (Cytiva). HCC1806 cells were grown in RPMI (Gibco) containing 10% FBS (Cytiva). BT20 human breast carcinoma cells were a gift from Dr Michael Lee (UMass Chan) and were grown in MEM (Gibco) containing 10% FBS (Cytiva). Cells were stimulated with human IGF-1 as described in figure legends (Sigma-Aldrich). All cells were screened regularly and tested negative for *mycoplasma* by PCR (Abm).

IRS2 (*IRS2*^*−/−*^) KO and control (NT) cells were generated by CRISPR/Cas9-mediated gene editing by electroporation of a ribonucleoprotein (RNP) complex of CRISPR-gRNA and Cas9 protein prepared according to manufacturer’s protocol (IDT). Cas9 used was Alt-R S.p. Cas9 Nuclease V3 (IDT). Guide RNA used: sgIRS2, CAA GGA CGA GTA CTT CGC CG (IDT Hs.Cas9.IRS2.1.AA). The control non-targeting guide RNA (sgNT) was purchased from IDT (Alt-R Cas9 Negative Control crRNA #1). Electroporation of ribonucleoprotein complex was done using nucleofector kit V (Lonza) and Nucleofector II electroporation machine (Amaxa, Program X-013 (SUM159PT and MDA-MB-231), Program T-020 (MCF10 A)).

All knock-down experiments were conducted with DharmaFECT one transfection reagent and siRNA from Horizon Discovery. siRNA-A negative (scrambled) control was purchased from Santa Cruz. *PIK3CA* knock-down was achieved with ON-TARGETplus Human *PIK3CA* (5290) siRNA – SMARTpool. *PIK3CA* siRNA was prepared according to the manufacturer’s protocol. Cells were incubated with *PIK3CA* siRNA at the indicated concentrations for 72 h prior to measurement of cell viability or preparation of protein lysate. *FOXO1* knock-down was achieved with ON-TARGETplus Human *FOXO1* (2308) siRNA – SMARTpool. *FOXO3* knock-down was achieved with ON-TARGETplus Human *FOXO3* (2309) siRNA – SMARTpool. *FOXO1* and *FOXO3* siRNAs were prepared according to the manufacturer’s protocol. Cells were incubated with siRNA for 48 h prior to the addition of dimethyl sulfoxide (DMSO) or alpelisib for 24 additional hours (final concentration DMSO 0.1%, alpelisib 5 μM). *FOXO1* and *FOXO3* individual knock-down was achieved with 50 nM siRNA for respective targets. *FOXO1* and *FOXO3* double knock-down was achieved with 50 nM siRNA for *FOXO1* and 50 nM siRNA for *FOXO3*. siRNA concentration was calculated using culture volume prior to the addition of DMSO or alpelisib.

### Plasmids

Human IRS1 and IRS2 were a kind gift from Adrian Lee (University of Pittsburgh). cDNA was subcloned into the pCDH-CMV-MCS-EF1-puro lentiviral vector (System Bioscience) with a C-terminal FLAG tag (84). The IRS2-Y5F mutant construct was generated by Genewiz and subcloned into the pCDH-CMV-MCS-EF1-puro lentiviral vector (System Bioscience) with a C-terminal FLAG tag (60). EV control plasmid was the pCDH-CMV-MCS-EF1-puro lentiviral vector (System Bioscience). Lentiviruses were generated by cotransfection of IRS1 or IRS2 plasmids and packaging plasmids (pMD2.G and psPAX2) into HEK293FT cells using lipofectamine 3000 according to the manufacturer’s instruction (Invitrogen). Cells were infected for 24 h with virus in the presence of 8 μg/ml polybrene (Sigma-Aldrich) and selected with 2 μg/ml puromycin (Gold Biotechnology).

### Drugs

Alpelisib was prepared at 150 mM in DMSO prior to diluting for experiments (MedChemExpress). MK2206 was prepared at 25 mM in DMSO prior to diluting for experiments (MedChemExpress).

### Crystal violet viability assay

Cells were split on day 1, then trypsinized, counted, and plated in 96 well plates on day 2. Cells were not plated in perimeter wells; these were filled with PBS. On day 3, media was removed, and fresh media containing drugs was added. Drug doses were selected to achieve a complete viability curve so that IC50 could be accurately determined, with two doses lower than the linear portion of the curve and two doses higher than the linear portion of the curve (56). The alpelisib doses used are clinically relevant, as median plasma concentrations of alpelisib stay above 2.26 μM (1 μg/ml) in humans for at least 8 h following a single 270 mg dose of alpelisib (FDA approved dose is 300 mg) (https://www.fda.gov/drugs/resources-information-approved-drugs/fda-approves-alpelisib-metastatic-breast-cancer) (85). Plates were incubated for 72 h, then media was removed and cells were fixed in paraformaldehyde (4% in PBS) for 40 min (Boston Bioproducts). Paraformaldehyde was removed and cells were stained with crystal violet (0.2%) for 70 min (Sigma-Aldrich). Following staining, wells were washed three times with water and plates were allowed to dry. Once plates were dry, 1% SDS was added to the wells and plates were agitated for 30 min to dissolve crystal violet. Absorbance was measured using a microplate reader (Promega GloMax Explorer, software v.4.0.0., absorbance 600 nm).

### FLICK viability assay

The FLICK assay was performed as described previously (53). Briefly, cells were incubated with increasing doses of drug and a fixed concentration of SYTOX Green (Invitrogen). Plates were either lysed at the start of the experiment, or were read throughout the drug treatment time course, then lysed and read after 72 h of drug treatment. Plates were lysed with 0.1% Triton X-100 (final concentration) (BioRad). Black-walled, optical bottom, 96-well microplates were used for these experiments (Corning, Greiner Bio-One, Thermo Fisher Scientific). Plates were read with a microplate reader (Ex: 485 nm/20 nm, Em: 528 nm/20 nm) (BioTek Synergy HT microplate reader, software v.3.11.19. Optics: Bottom, Gain: 55, Read speed: normal). This microplate reader was suitable for the FLICK assay because the gain can be set manually, which is crucial for accuracy in repeated measurements of the same plate throughout a time course.

### Immunoblotting

For whole-cell extracts, cells were solubilized at 4 °C in lysis buffer (20 mM Tris buffer, pH 7.4, 1% Nonidet P-40, 0.137 M NaCl, 10% glycerol) containing phosphatase inhibitors (Roche) and protease inhibitors (Roche). Cell extracts containing equivalent amounts of protein were resolved by SDS-PAGE and transferred to nitrocellulose membranes. Membranes were blocked using 5% nonfat milk in 1x Tris-buffered saline with Tween-20 (TBST) (50 mM Tris, pH 7.5, 150 mM NaCl, 0.01% Tween 20). Primary antibody incubations were carried out in 5% bovine serum albumin or 5% nonfat milk in 1X TBST overnight at 4 °C. Primary antibodies used in this study include: IRS1 (Cell Signaling Technology [CST] #95816; Bethyl Laboratories #A301 to 158A, sold by Thermo Fisher Scientific), IRS2 (CST #4502), GAPDH (Santa Cruz Biotechnology #sc-32233), phospho-AKT S473 (CST #9271), AKT (CST #9272), α-tubulin (CST #3873), phospho-ERK (CST #9101), ERK (CST #9102), Actin (Invitrogen # MA5-15739), FOXO1 (CST #2880), FOXO3a (CST #12829), and Lamin A/C (Santa Cruz Biotechnology #sc-376248). Secondary antibody (Jackson ImmunoResearch Peroxidase AffiniPure Goat Anti-Rabbit IgG (H + L) #111-035 to 144 and Goat Anti-Mouse IgG (H + L) #115–035 to 146) incubations were carried out in 5% nonfat milk in 1X TBST for 1 h at RT. Bands were detected by chemiluminescence using a ChemiDoc XRS + system (Bio-Rad), using Clarity Western ECL Substrate (BioRad) or SuperSignal West Femto Maximum Sensitivity Substrate (Thermo Fisher Scientific).

For immunoblot quantification, the signals from the protein of interest were normalized with that of the loading control, and the signals from phospho-specific antibodies were normalized to the total protein of interest using Image J (v.1.52) or BioRad Image Lab (v.6.1.0 build 7). Only bands that produced a signal which did not saturate the ChemiDox XRS + detector were used for quantification. All immunoblotting images used for quantification were 16 bit TIFF files.

### Nuclear/cytoplasmic fractionation

Nuclear and cytoplasmic extraction was carried out using the NE-PER Nuclear and Cytoplasmic Extraction Reagents (Thermo Fisher Scientific) according to manufacturer’s protocol, with one modification. Following cytoplasmic extraction, (nuclear) pellets were washed once with cold 1X PBS, briefly resuspended, then centrifuged at maximum speed in a microcentrifuge for 1 min. PBS was then completely removed prior to resuspending the pellets in ice-cold nuclear extraction reagent. Extracts were then analyzed by immunoblotting as described.

### qPCR

RNA was extracted using the RNA-easy kit (Qiagen). cDNA was synthesized using an AzuraQuant cDNA synthesis kit (Azura Genomics). qPCR was performed in an Applied Biosystems QuantStudio 6 Flex apparatus using AzuraView GreenFast qPCR Blue Mix (Azura Genomics). The delta-delta Ct method was used to determine relative mRNA expression (r18s used for normalization) (86). Primers used were as follows: h*IRS1*-Fwd 5′-CAA CTG GAC ATC ACA GCA GAA-3′; h*IRS1*-Rev 5′-ACT GAA ATG GAT GCA TCG TAC C-3′; h*IRS2*-Fwd 5′-CCA CCA TCG TGA AAG AGT GA-3′; h*IRS2*-Rev 5′-CAG AGT CCA CAG ATG TTT CCA A-3′; r18s-Fwd 5′-GTC GCT CGC TCC TCT CCT ACT-3′; r18s-Rev 5′-TCT GAT AAA TGC ACG CAT CCC-3′. The average delta Ct for the control condition (WT or WT treated with DMSO) across all replicates was used to calculate delta-delta Ct values. This enables calculation of relative mRNA expression values for all conditions and replicates while also retaining and displaying the variability in the control condition for all replicates. 2^-ΔΔCT^ (relative expression) was then calculated for all conditions and replicates. All relative expression values were then normalized to the average of the three control condition relative expression values so that the mean of the controls was equal to 1.

### ChIP qPCR

Prior to ChIP, cells were treated with either DMSO or alpelisib (5 μM) for 24 h. ChIP was carried out with the ChIP-IT Express Chromatin Immunoprecipitation Kit according to the manufacturer’s protocol (Active Motif) using the following antibodies: IgG control (CST #2729), FOXO1 (CST #2880), and FOXO3a (CST #12829). The qPCR primers used to detect FOXO1/3 binding to the human IRS2 promoter were Fwd 5′-CTG TGT GTG CCT GCG TAA-3′; Rev 5′-CCG CAC AGT GAG TAA CAC AT-3′.

### Bioinformatics and statistical analysis

#### Clinical data in violin plots

Clinical data shown in Figure 1, *A* and *B*, Figure S1, *A*–*E*, and Figure S3*A* were derived from the Molecular Taxonomy of Breast Cancer International Consortium dataset, accessed using the cBioPortalData (version 2.16.0) R package (36, 87). Gene expression measurements (log2 intensity values) derived from the Illumina HT-12 v3 microarray platform were retrieved for 1980 total breast cancer samples. Samples were grouped according to *PIK3CA* genomic status into mutant (n = 795) and WT (n = 1185) cohorts. Breast cancer subtypes were defined as Luminal A&B (ER+ and HER2-), Triple-negative (ER-, PR-, HER2-), and HER2 enriched (HER2+) (ER = estrogen receptor. HER2 = human epidermal growth factor receptor 2) (http://www.ncbi.nlm.nih.gov/books/NBK583808/). Data shown in Figure 1, *C* and *D* were derived from the Clinical Proteomic Tumor Analysis Consortium dataset, accessed *via* https://kb.linkedomics.org/ (40). For each gene, expression distributions were assessed for normality using the Shapiro–Wilk test (88). Group-wise expression patterns were visualized using combined violin and box plots generated with ggplot2(version 3.5.2) and ggpubr(version 0.6.1) (89) (https://rpkgs.datanovia.com/ggpubr/) Statistical comparisons between *PIK3CA* mutant and WT groups were performed using the Wilcoxon rank-sum test (90). All statistical analyses and figure generation were performed in R (version 4.4.1) (https://cir.nii.ac.jp/crid/1574231874043578752).

#### DepMap-derived data

Data shown in Figure 2*G* were from Batch corrected Expression Public 24Q2 dataset (DepMap: The Cancer Dependency Map Project at Broad Institute, depmap.org) (41). Data shown in Figure 2*H* were from RPPA500 MCLP (DepMap). For Figure 8, data shown is from all cell lines, excluding myeloid and lymphoid-derived cell lines. All mRNA data (Fig. 8, *A* and *C*, *D*, Fig. S7, *A* and *B*) is from Expression Public 25Q3 (IRS1 or IRS2 log2(TPM+1)). Protein expression for Figure 8*B* is from Relative Protein Expression Harmonized MS CCLE Gygi (DepMap). Drug sensitivity association results for all panels of Figure 8 and Figure S7 are from Sanger Genomics of Drug Sensitivity in Cancer 2 (GDSC2) (1053 - MK2206, 1560 - alpelisib)(accessed using DepMap) (61, 62). In Figure 8, *C* and *D*, only drugs that specifically inhibited PI3K or AKT (and did not inhibit other kinases) were labeled as PI3K/AKT inhibitors and were colored pink or purple. PI3K and AKT isoform-specific inhibition for drugs labeled PI3K/AKT inhibitors was determined according to prior literature (91, 92). All volcano plot analyses and figures were generated in R (version 4.4.1) using ggplot2(version 3.5.2) for plotting and ggrepel(version 0.9.6) for nonoverlapping labels (89) (https://cir.nii.ac.jp/crid/1574231874043578752, https://slowkow.com/projects/ggrepel/).

#### Statistical analysis of cell viability

IC50 values reported throughout the paper are relative IC50 values. IC50 values and *p* values from comparison of IC50 values are only provided when sufficient data are present to accurately estimate IC50 values. Mathematical modeling shows that a cell viability curve requires at least two drug concentrations below and two drug concentrations above the linear portion of the curve (the straight line that includes the IC50) to accurately calculate the IC50 and therefore to accurately compare IC50 values. For graphs that do not fit these criteria, IC50 values and IC50 comparison *p* values are not listed because these values, while sometimes possible to calculate, are likely inaccurate (56). In these cases, AUC was used to determine if LF results were statistically significantly different (GraphPad Prism). AUCs were compared using Welch’s *t* test (two-tailed), utilizing AUC total area and standard error.

#### Other statistical analysis and software

Statistical analysis and graphing was performed using GraphPad Prism (Version 10.6.1) for all panels except Figure 1, *A*–*D*, Figure S1, *A*–*E*, Figure S3*A*, and Figure 8, *C* and *D*. Specific statistical tests applied for each dataset are described in the corresponding Figure Legends. Additional detail for Figure 6*C*, Figure 7*C*, and Figure S6*A*: Protein phosphorylation ratios and total protein abundances were log-transformed using the natural logarithm prior to analysis. The primary statistical model was an ordinary least squares ANOVA applied to log-transformed ratios, with fixed effects for genotype, dose (treated as a categorical factor with levels 0, 0.5, 1, and 2.5), their interaction, and a blocking factor for gel: log(ratio) ∼ genotype × dose + gel. Prespecified contrasts compared each genotype pair within every dose level on the log scale. Estimated marginal means and contrasts were obtained using the *emmeans* package, averaging over gel with equal weights. Inference was performed on the log scale. Because contrasts were planned *a priori* at specific doses, no multiplicity adjustment was applied. Model assumptions were assessed using Levene’s test across genotype × dose, the Breusch–Pagan test on residuals, and the Shapiro–Wilk test on standardized residuals. To address model heteroscedasticity in the primary analysis, *p* values were computed using HC3 robust standard errors. When the normality assumption was violated, we performed an additional predefined sensitivity analysis using the Aligned Rank Transform (ART) approach. ART preserves the factorial structure and provides valid inference under non-normality. ART ANOVA and planned contrasts were computed using the *ARTool* package in R(98). Analyses were conducted in R version 4.5.1 (aarch64-apple-darwin20) using the packages *dplyr*, *emmeans*, *car*, *lmtest*, and *sandwich*. BioRender was used to generate Figure 8*E*.

## Data availability

All data are contained within the article.

## Supporting information

This article contains supporting information.

## Conflict of interest

The authors declare that they have no conflicts of interest with the contents of this article.
