# Supplementary material for: Insulin receptor substrate 2 (IRS2) confers resistance to PI3K pathway inhibition in PIK3CA mutant breast cancer
Source: J Biol Chem. 2026 Aug 10;302(10):113426. doi: 10.1016/j.jbc.2026.113426 (PMC13571430; doi:10.1016/j.jbc.2026.113426)
Supplement: Supplementary file 1 [file mmc1.pdf]

Figure S1

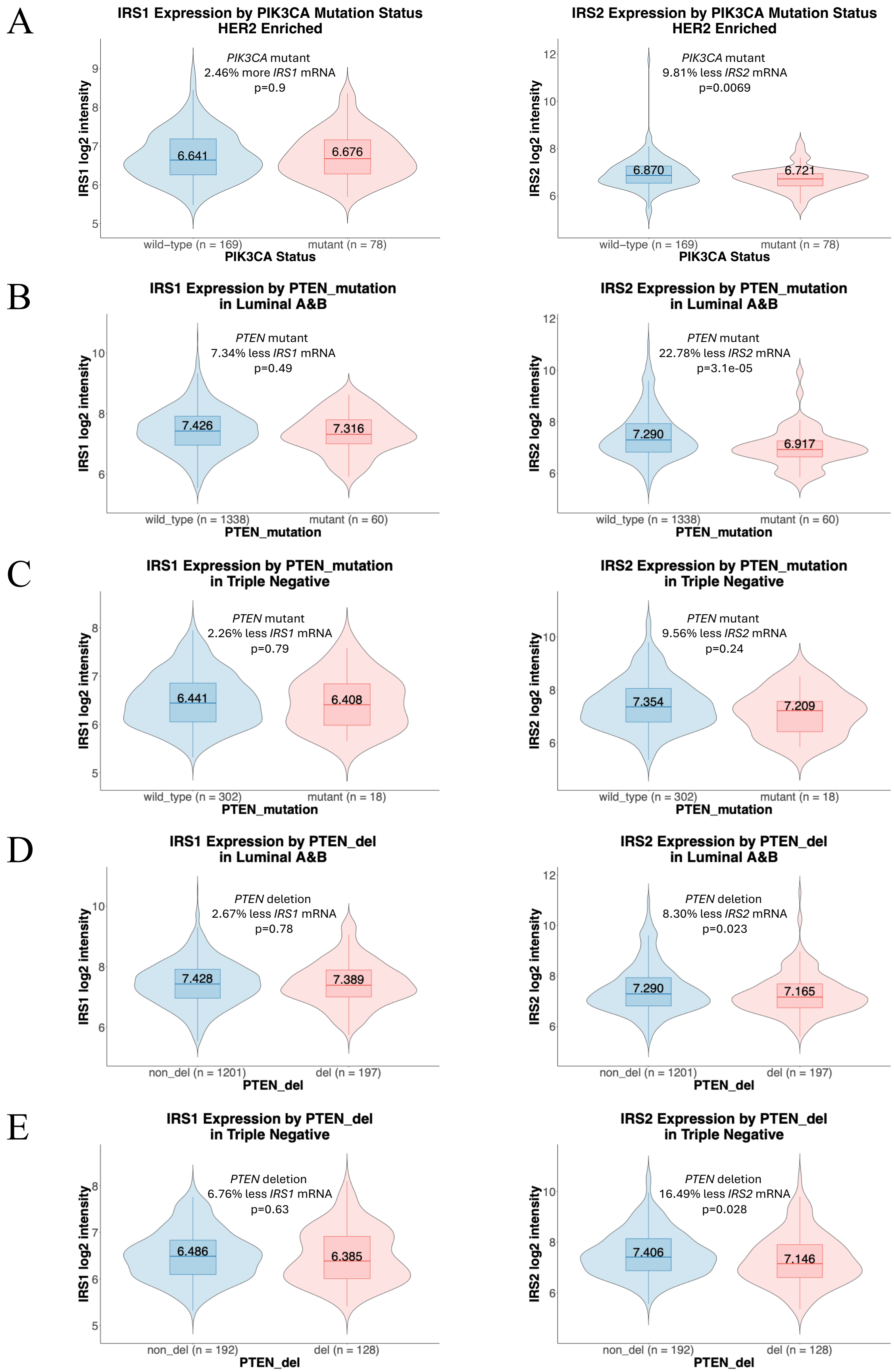

A
IRS1 Expression by PIK3CA Mutation Status
HER2 Enriched
PIK3CA mutant
2.46% more IRS1 mRNA
p=0.9
IRS1 log2 intensity
6.641
6.676
wild−type (n = 169)
mutant (n = 78)
PIK3CA Status
IRS2 Expression by PIK3CA Mutation Status
HER2 Enriched
PIK3CA mutant
9.81% less IRS2 mRNA
p=0.0069
IRS2 log2 intensity
6.870
6.721
wild−type (n = 169)
mutant (n = 78)
PIK3CA Status
B
IRS1 Expression by PTEN_mutation
in Luminal A&B
PTEN mutant
7.34% less IRS1 mRNA
p=0.49
IRS1 log2 intensity
7.426
7.316
wild_type (n = 1338)
mutant (n = 60)
PTEN_mutation
IRS2 Expression by PTEN_mutation
in Luminal A&B
PTEN mutant
22.78% less IRS2 mRNA
p=3.1e-05
IRS2 log2 intensity
7.290
6.917
wild_type (n = 1338)
mutant (n = 60)
PTEN_mutation
C
IRS1 Expression by PTEN_mutation
in Triple Negative
PTEN mutant
2.26% less IRS1 mRNA
p=0.79
IRS1 log2 intensity
6.441
6.408
wild_type (n = 302)
mutant (n = 18)
PTEN_mutation
IRS2 Expression by PTEN_mutation
in Triple Negative
PTEN mutant
9.56% less IRS2 mRNA
p=0.24
IRS2 log2 intensity
7.354
7.209
wild_type (n = 302)
mutant (n = 18)
PTEN_mutation
D
IRS1 Expression by PTEN_del
in Luminal A&B
PTEN deletion
2.67% less IRS1 mRNA
p=0.78
IRS1 log2 intensity
7.428
7.389
non_del (n = 1201)
del (n = 197)
PTEN_del
IRS2 Expression by PTEN_del
in Luminal A&B
PTEN deletion
8.30% less IRS2 mRNA
p=0.023
IRS2 log2 intensity
7.290
7.165
non_del (n = 1201)
del (n = 197)
PTEN_del
E
IRS1 Expression by PTEN_del
in Triple Negative
PTEN deletion
6.76% less IRS1 mRNA
p=0.63
IRS1 log2 intensity
6.486
6.385
non_del (n = 192)
del (n = 128)
PTEN_del
IRS2 Expression by PTEN_del
in Triple Negative
PTEN deletion
16.49% less IRS2 mRNA
p=0.028
IRS2 log2 intensity
7.406
7.146
non_del (n = 192)
del (n = 128)
PTEN_del

Figure S2

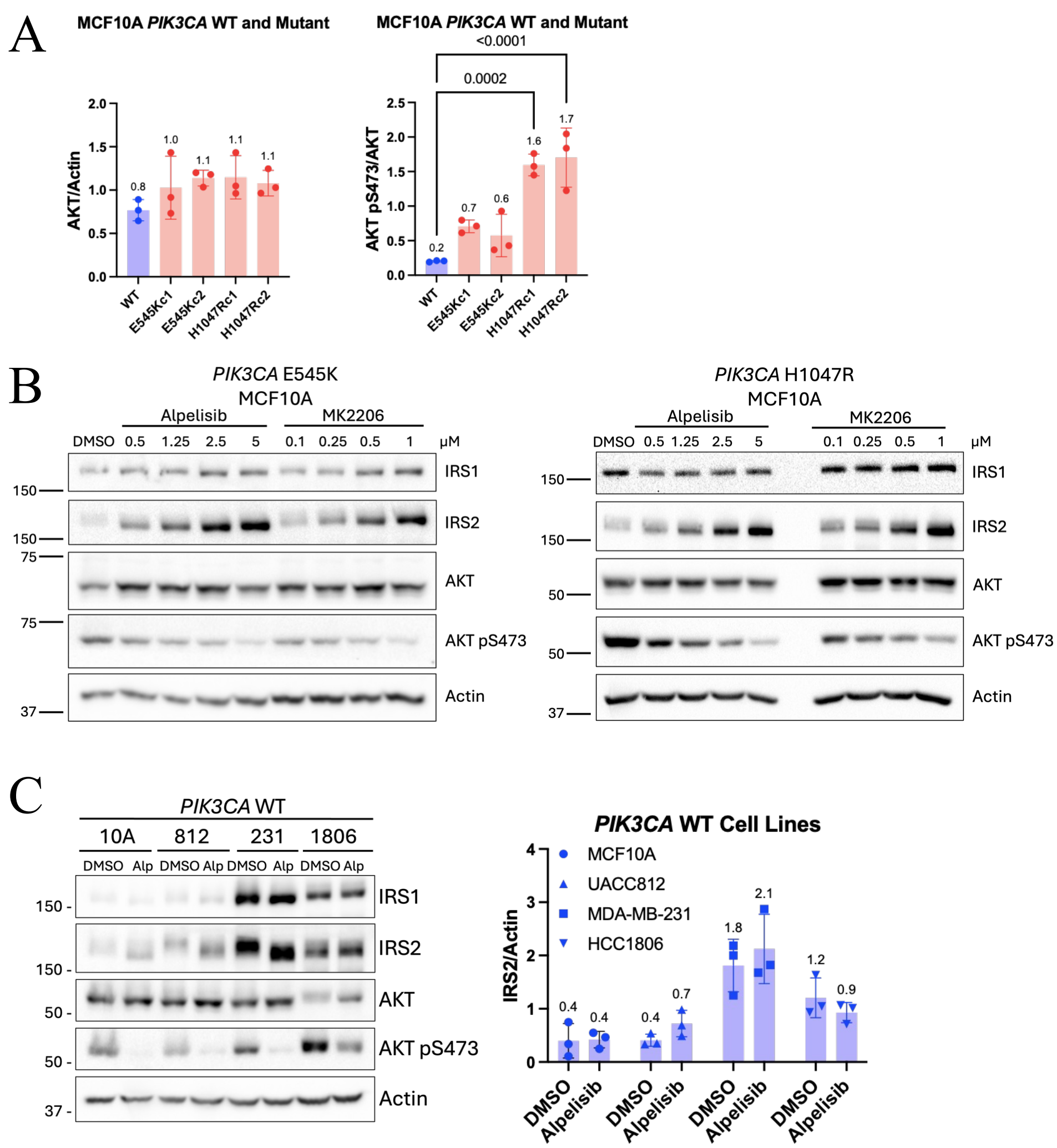

A
MCF10A PIK3CA WT and Mutant
AKT/Actin
WT
E545Kc1
E545Kc2
H1047Rc1
H1047Rc2
AKT pS473/AKT
<0.0001
0.0002
B
PIK3CA E545K
MCF10A
Alpelisib
MK2206
DMSO 0.5 1.25 2.5 5 0.1 0.25 0.5 1 µM
IRS1
IRS2
AKT
AKT pS473
Actin
PIK3CA H1047R
MCF10A
C
PIK3CA WT
10A
812
231
1806
DMSO Alp DMSO Alp DMSO Alp DMSO Alp
PIK3CA WT Cell Lines
MCF10A
UACC812
MDA-MB-231
HCC1806
IRS2/Actin
DMSO
Alpelisib

Figure S3

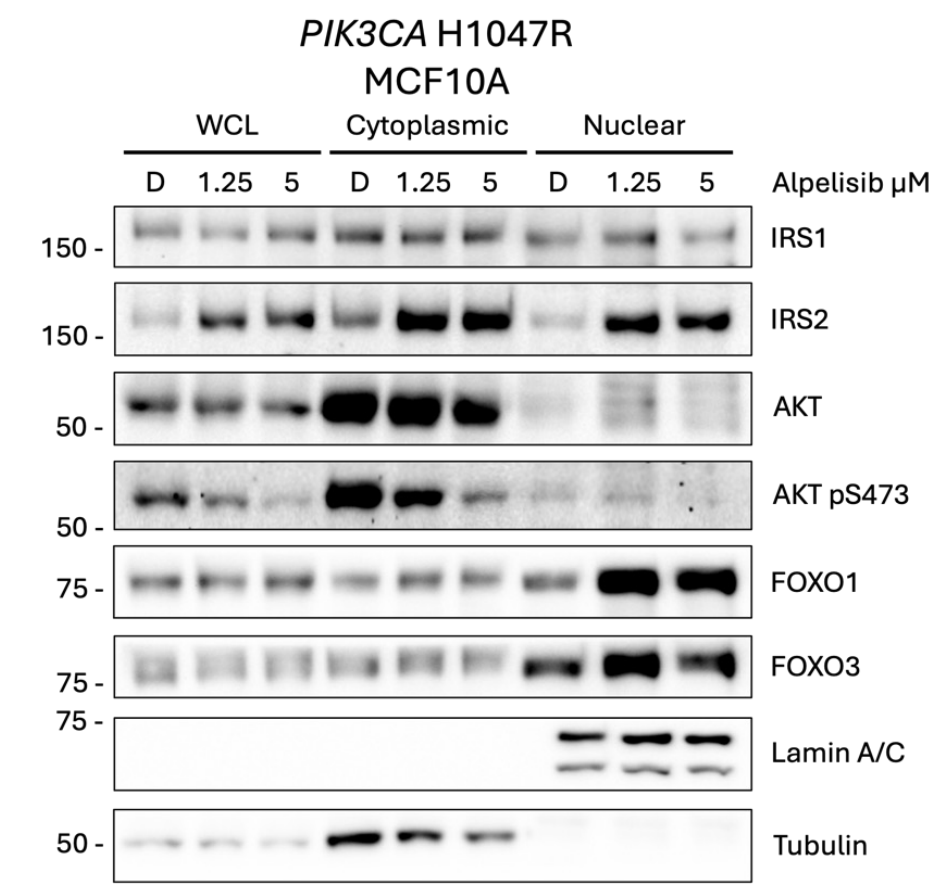
PIK3CA H1047R
MCF10A
WCL
Cytoplasmic
Nuclear
D
1.25
5
D
1.25
5
D
1.25
5
Alpelisib μM
150 -
IRS1
150 -
IRS2
50 -
AKT
50 -
AKT pS473
75 -
FOXO1
75 -
FOXO3
75 -
Lamin A/C
50 -
Tubulin

Figure S4

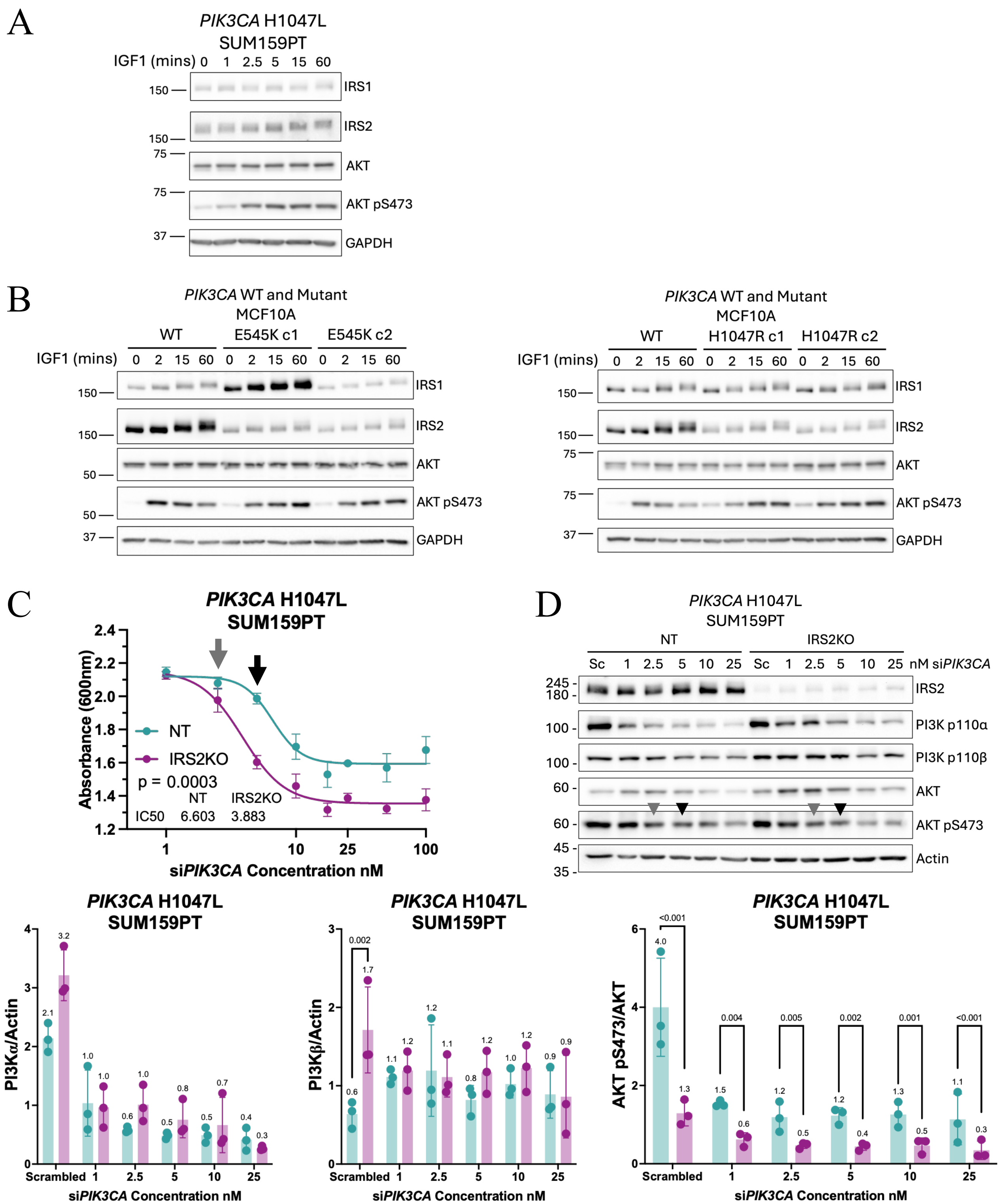
A
PIK3CA H1047L
SUM159PT
IGF1 (mins) 0 1 2.5 5 15 60
IRS1
IRS2
AKT
AKT pS473
GAPDH
B
PIK3CA WT and Mutant
MCF10A
WT E545K c1 E545K c2
IGF1 (mins) 0 2 15 60 0 2 15 60 0 2 15 60
IRS1
IRS2
AKT
AKT pS473
GAPDH
PIK3CA WT and Mutant
MCF10A
WT H1047R c1 H1047R c2
IGF1 (mins) 0 2 15 60 0 2 15 60 0 2 15 60
C
PIK3CA H1047L
SUM159PT
Absorbance (600nm)
NT
IRS2KO
p = 0.0003
NT IRS2KO
IC50 6.603 3.883
siPIK3CA Concentration nM
D
PIK3CA H1047L
SUM159PT
NT IRS2KO
Sc 1 2.5 5 10 25 Sc 1 2.5 5 10 25 nM siPIK3CA
IRS2
PI3K p110α
PI3K p110β
AKT
AKT pS473
Actin
PIK3CA H1047L
SUM159PT
PI3Kα/Actin
Scrambled 1 2.5 5 10 25
siPIK3CA Concentration nM
PIK3CA H1047L
SUM159PT
PI3Kβ/Actin
0.002
Scrambled 1 2.5 5 10 25
siPIK3CA Concentration nM
PIK3CA H1047L
SUM159PT
AKT pS473/AKT
<0.001 0.004 0.005 0.002 0.001 <0.001
Scrambled 1 2.5 5 10 25
siPIK3CA Concentration nM

Figure S5

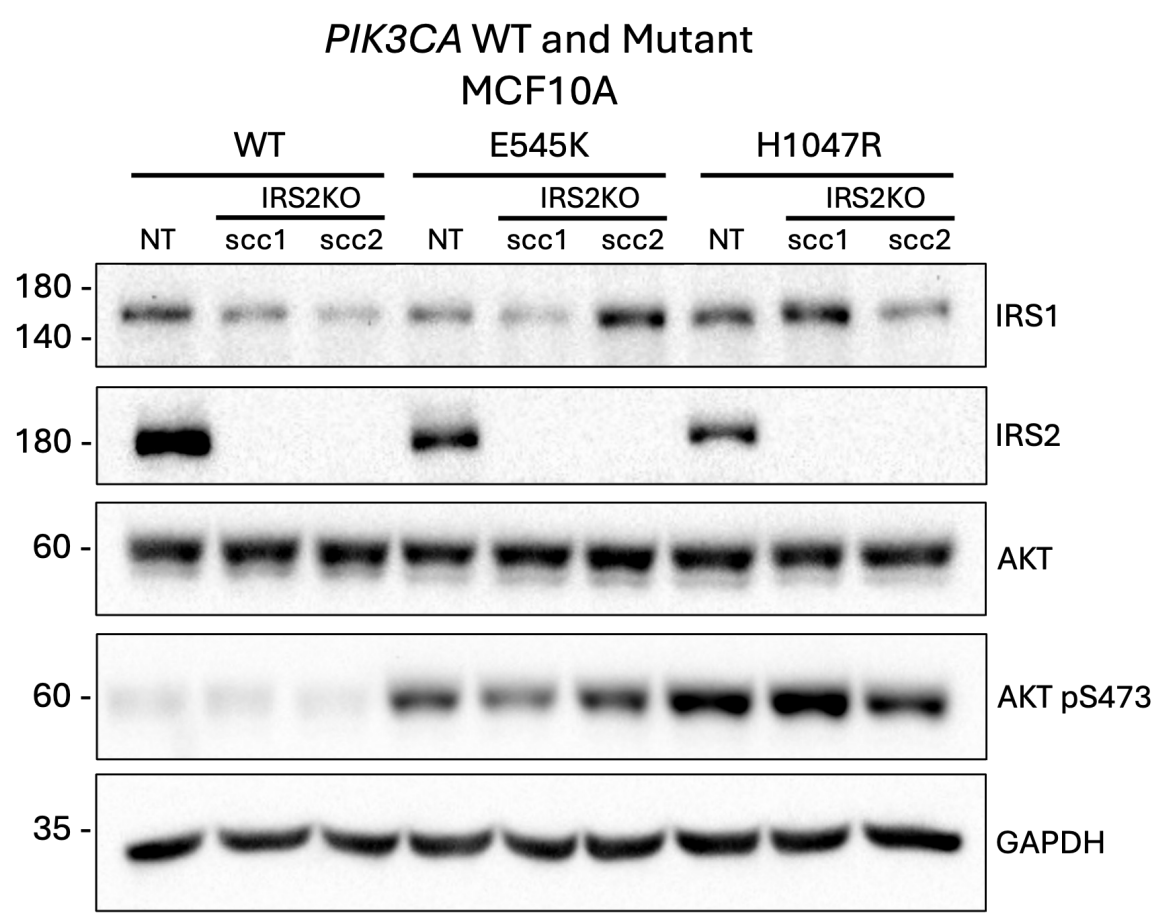

PIK3CA WT and Mutant
MCF10A
WT
E545K
H1047R
IRS2KO
NT
scc1
scc2
180 -
140 -
IRS1
180 -
IRS2
60 -
AKT
60 -
AKT pS473
35 -
GAPDH

Figure S6

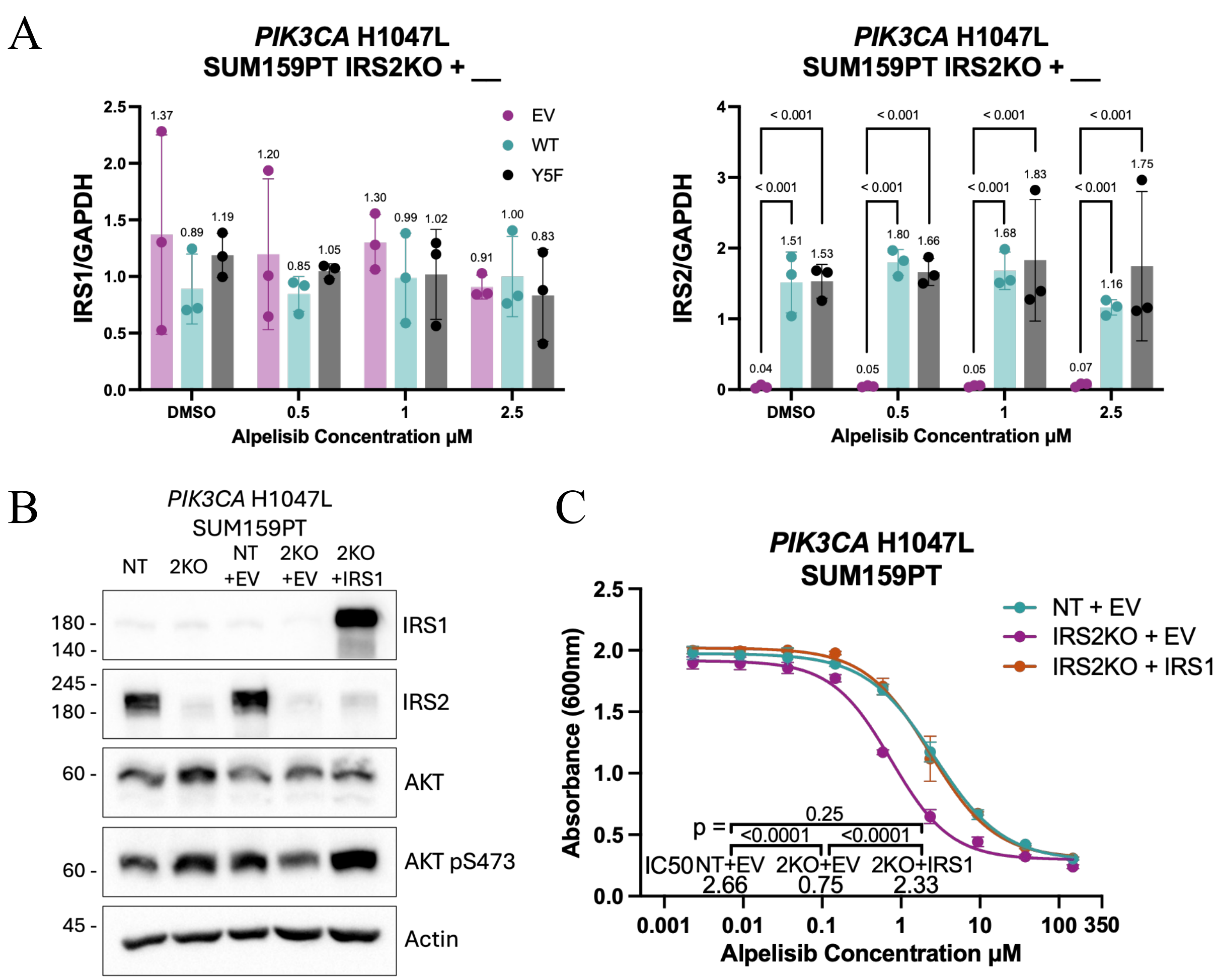

A
PIK3CA H1047L
SUM159PT IRS2KO + __
IRS1/GAPDH
EV
WT
Y5F
DMSO
0.5
1
2.5
Alpelisib Concentration μM
IRS2/GAPDH
< 0.001
B
PIK3CA H1047L
SUM159PT
NT
2KO
NT +EV
2KO +EV
2KO +IRS1
IRS1
IRS2
AKT
AKT pS473
Actin
C
PIK3CA H1047L
SUM159PT
NT + EV
IRS2KO + EV
IRS2KO + IRS1
Absorbance (600nm)
p =
0.25
<0.0001
<0.0001
IC50 NT+EV 2KO+EV 2KO+IRS1
2.66 0.75 2.33
Alpelisib Concentration μM

Figure S7

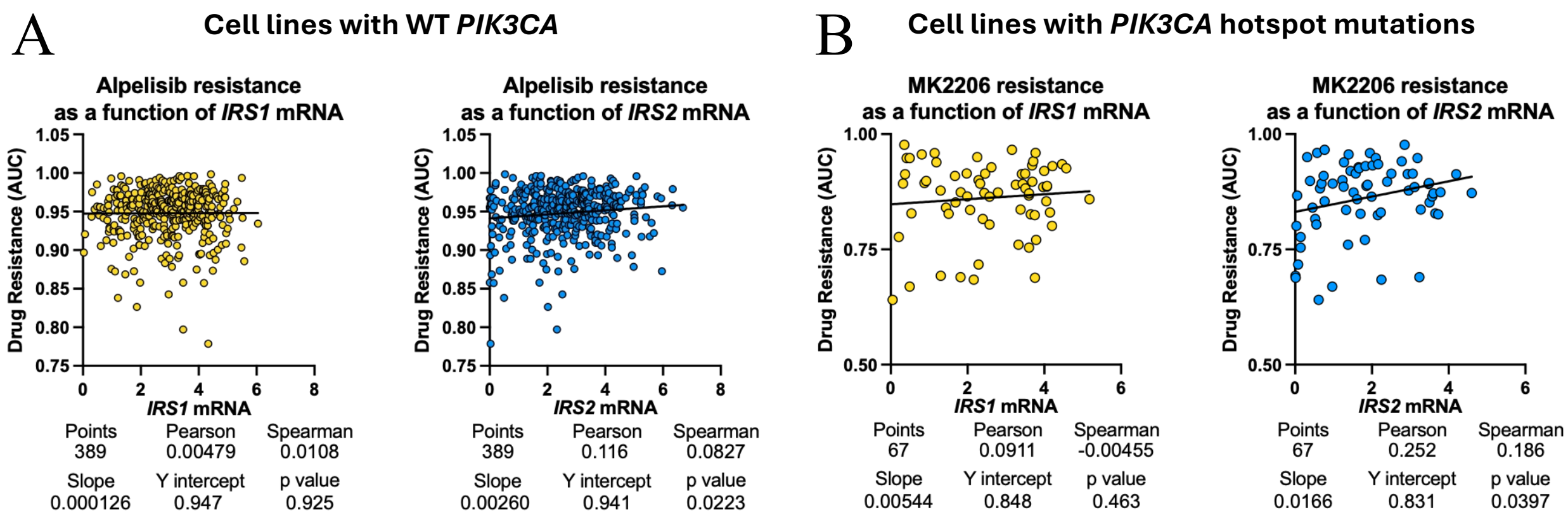
A
Cell lines with WT PIK3CA
Alpelisib resistance as a function of IRS1 mRNA
Drug Resistance (AUC)
IRS1 mRNA
Points 389
Pearson 0.00479
Spearman 0.0108
Slope 0.000126
Y intercept 0.947
p value 0.925
Alpelisib resistance as a function of IRS2 mRNA
Drug Resistance (AUC)
IRS2 mRNA
Points 389
Pearson 0.116
Spearman 0.0827
Slope 0.00260
Y intercept 0.941
p value 0.0223
B
Cell lines with PIK3CA hotspot mutations
MK2206 resistance as a function of IRS1 mRNA
Drug Resistance (AUC)
IRS1 mRNA
Points 67
Pearson 0.0911
Spearman -0.00455
Slope 0.00544
Y intercept 0.848
p value 0.463
MK2206 resistance as a function of IRS2 mRNA
Drug Resistance (AUC)
IRS2 mRNA
Points 67
Pearson 0.252
Spearman 0.186
Slope 0.0166
Y intercept 0.831
p value 0.0397
