# Supplementary material for: Insulin receptor substrate 2 (IRS2) confers resistance to PI3K pathway inhibition in PIK3CA mutant breast cancer
Source: J Biol Chem. 2026 Aug 10;302(10):113426. doi: 10.1016/j.jbc.2026.113426 (PMC13571430; doi:10.1016/j.jbc.2026.113426)
Supplement: Supplementary file 2 [file mmc2.pdf]

Supplemental Figure Legends

Figure S1. Reduction of *IRS2* mRNA abundance in *PIK3CA* mutant and *PTEN* altered breast cancer. (A) Differential gene expression analysis of *IRS1* and *IRS2* mRNA in *PIK3CA* WT and *PIK3CA* mutant HER2-enriched breast tumors in the METABRIC dataset. (B and C) Differential gene expression analysis of *IRS1* and *IRS2* mRNA in *PTEN* WT and *PTEN* mutant luminal A&B (B) and triple-negative (C) breast cancer (METABRIC dataset). (D and E) Differential gene expression analysis of *IRS1* and *IRS2* mRNA in *PTEN* non-deleted (non_del) and *PTEN* deleted (del) luminal A&B (D) and triple-negative (E) breast cancer (METABRIC dataset). Gene expression measurements shown as log2 intensity values (Illumina HT-12 v3 microarray). For all violin plots, box and whisker plots show the median, 25%(Q1), 75%(Q3) of data (box), Q1 - 1.5 × interquartile range (IQR), and Q3 + 1.5 × IQR of data (whiskers). For all panels, percent changes were determined by comparing the medians of the original data (prior to log transformation). For all panels, statistical analysis by Wilcoxon rank sum test with continuity correction.

Figure S2. *PIK3CA* mutation reversibly suppresses IRS2 mRNA and protein abundance in mammary epithelial cells and breast cancer cell lines. (A) *PIK3CA* WT and *PIK3CA* mutant knockin MCF10A cells were evaluated for AKT and pS473-AKT expression (see Figure 2B). The data shown represent the mean +/- SD of three independent experiments. Statistical analysis by One-Way ANOVA followed by Dunnett's multiple comparisons test to compare mutants against WT (P values shown). (B) *PIK3CA* E545K and *PIK3CA* H1047R mutant knockin MCF10A cells were treated with increasing concentrations of alpelisib or MK2206 for 24 hrs and cell extracts were evaluated by immunoblot. (C) *PIK3CA* WT breast epithelial and cancer cell lines were treated with or without alpelisib (5µM) for 24 hrs and cell extracts were evaluated by immunoblot. The data shown represent the mean +/- SD of three independent experiments. Statistical analysis by

Two-Way ANOVA. Because these comparisons were planned a priori, no multiplicity adjustments were applied (P values shown). (10A - MCF10A, 812 - UACC812, 231 – MDA-MB-231, 1806 - HCC1806).

Figure S3. Mutant PIK3CA regulates IRS2 expression through FOXO1 and FOXO3. *PIK3CA* mutant H1047R MCF10A cells were treated in plain media with or without alpelisib for 24 hours and cytoplasmic and nuclear extracts were evaluated by immunoblot (D = DMSO).

Figure S4. IGF1 stimulation increases PI3K pathway signaling in *PIK3CA* mutant breast epithelial and cancer cell lines. (A) SUM159PT cells (*PIK3CA* H1047L) and (B) *PIK3CA* mutant E545K and H1047R MCF10A cells were serum starved overnight, then treated with or without IGF1 (50ng/mL) for increasing amounts of time and cell extracts were evaluated by immunoblot. (C) Viability of *PIK3CA* mutant SUM159PT NT and IRS2KO cells treated with increasing concentrations of *PIK3CA* siRNA for 72 hrs, measured by crystal violet staining. Data shown are representative of three independent experiments. Statistical analysis by variable slope (four parameters) nonlinear regression using least squares regression to fit the curve (with asymmetrical confidence intervals calculated for parameters) and the extra sum-of-squares F test to compare IC50 values. (D) *PIK3CA* mutant SUM159PT NT and IRS2KO cells were treated with increasing concentrations of *PIK3CA* siRNA for 72 hrs and cell extracts were evaluated by immunoblot. The data shown in the graphs below represent the mean +/- SD of three independent experiments. (Sc = scrambled (negative control) siRNA). Statistical analysis was performed using a natural-log transformation followed by ordinary least squares (OLS) ANOVA on log(ratio), with fixed effects for genotype (NT vs IRS2KO), dose (categorical: Scrambled siRNA negative control, 1, 2.5, 5, 10, 25), their interaction, and a gel blocking factor, according to the model: $\log(\text{ratio}) \sim \text{genotype} \times \text{dose} + \text{gel}$. Pre-specified comparisons between NT and IRS2KO were conducted within each dose

on the log scale. Because these comparisons were planned a priori, no multiplicity adjustments were applied (P values shown).

Figure S5. IRS2 depletion reduces cell proliferation and increases cell death in *PIK3CA* mutant mammary epithelial cells in response to PI3K pathway inhibitors. (A) MCF10A *PIK3CA* WT and *PIK3CA* mutant cells were treated with non-targeting guide RNA (*NT*) or IRS2-targeting guide RNA (IRS2KO) and cell extracts were analyzed by immunoblot (see Figure 5).

Figure S6. IRS2-dependent PI3K activation confers resistance to PI3K inhibition in *PIK3CA* mutant breast cancer. (A) *PIK3CA* H1047L mutant SUM159PT IRS2KO cells expressing empty vector (EV), IRS2-WT or IRS2-Y5F were treated with increasing concentrations of alpelisib for 24 hrs and cell extracts were analyzed for IRS1 and IRS2 expression by immunoblot (see Figure 7C). The data shown in the graphs represent the mean +/- SD of three independent experiments. Statistical analysis was performed using a natural-log transformation followed by ordinary least squares (OLS) ANOVA on log(ratio), with fixed effects for genotype (EV vs. IRS2-WT vs. IRS2-Y5F), dose (categorical: 0 (DMSO), 0.5, 1, 2.5), their interaction, and a gel blocking factor, according to the model: log(ratio) ∼ genotype × dose + gel. Pre-specified comparisons between each genotype pair within each dose were conducted on the log scale. Because these comparisons were planned a priori, no multiplicity adjustments were applied (P values shown). (B) *PIK3CA* H1047L mutant SUM159PT NT and IRS2KO (2KO) cells expressing empty vector (EV) or IRS1-WT were analyzed by immunoblot. (C) Viability of *PIK3CA* H1047L mutant SUM159PT NT cells expressing empty vector (EV) and IRS2KO cells expressing empty vector (EV) or IRS1-WT treated with increasing concentrations of alpelisib for 72 hrs, measured by crystal violet staining. Data shown are representative of three independent experiments. Statistical analysis by variable slope (four parameters) nonlinear regression using least squares regression to fit the curve (with

asymmetrical confidence intervals calculated for parameters) and the extra sum-of-squares F test to compare IC50 values.

Figure S7. IRS2 expression positively correlates with resistance to PI3K pathway inhibitors in *PIK3CA* mutant cancer cell lines. (A) Correlation between *IRS1* and *IRS2* mRNA and sensitivity to alpelisib in cancer cell lines with WT *PIK3CA* (excluding myeloid and lymphoid-derived cell lines) in the DepMap dependency dataset. (B) Correlation between *IRS1* and *IRS2* mRNA and sensitivity to MK2206 in cancer cell lines with *PIK3CA* hotspot mutations (excluding myeloid and lymphoid-derived cell lines) in the DepMap dependency dataset. For (A) and (B), P values shown are from simple linear regression, $p<0.05$ indicates that the slope is significantly different from zero. mRNA abundance shown is log2(TPM+1).
